# A randomised control trial study evaluating a compulsive exercise group for adolescent inpatients with eating disorders

**DOI:** 10.1007/s40519-025-01809-1

**Published:** 2025-12-17

**Authors:** Eleanor Herrmann, Amie Garghan, Gurdeep Aulakh, Natasha Cogings, Pria Sandhu, Jessica Grant, Josephine Greenhill, J. Hubert Lacey

**Affiliations:** 1 Schoen Clinic Newbridge, Birmingham, UK; 2 City St George’s, University of London, London, UK

**Keywords:** Anorexia nervosa, Compulsive exercise, Group therapy, CBT, Psychoeducation

## Abstract

**Purpose:**

To evaluate the efficacy of a 7-session manualised group intervention for Compulsive Exercise (NEAT) as an adjuvant to inpatient treatment for adolescents with Anorexia Nervosa (AN).

**Methods:**

Adolescents aged 12–17 consented to the study (*N* = 75). Using a randomised control design, they were allocated to the NEAT group with treatment as usual (NEAT + TAU) (*N* = 43), or to treatment as usual alone (TAU) (*N* = 32). Self-report measures of compulsive exercise and eating disorder psychopathology were administered at two timepoints to assess the efficacy of the intervention.

**Results:**

51 participants completed the study (NEAT + TAU *N* = 27; TAU *N* = 24). Both groups saw a significant decrease in compulsive exercise and eating disorder psychopathology between timepoints. There were no significant differences between treatment groups on the outcome measures.

**Conclusions:**

Intensive, multidisciplinary TAU, and NEAT group added to TAU were effective at reducing compulsive exercise and eating disorder symptoms. Clinical opinion and recommendations for further study are discussed. Treatment manual is made available below.

*Level of evidence*: Level I Evidence obtained from: at least one properly designed randomised controlled trials; systematic reviews and meta-analyses; experimental studies.

**Supplementary Information:**

The online version contains supplementary material available at 10.1007/s40519-025-01809-1.

## Introduction

Compulsive exercise is a clinical term describing exercise driven by fear of weight or weight gain or to regulate emotions, often negatively impacting physical and mental well-being [1, 2]. It is a common feature of most eating disorders, particularly anorexia nervosa (AN) [3], and plays a significant role in both the development and maintenance of AN [4–6]. Among young individuals with eating disorders, compulsive exercise is the most prevalent weight-control behaviour and has been suggested as a potential gateway to further eating disordered behaviours, such as purging [7]. Research in adult populations with AN indicates that compulsive exercise is associated with greater illness severity [3], prolonged inpatient admissions [8], and an increased risk of relapse [9].

A 2017 editorial [10] highlighted the need for treatment to address compulsive exercise in adolescents with eating disorders. Despite its high prevalence and detrimental effects, there remains limited guidance on effective interventions. Notably, the NICE Guidelines, the UK official guidance to treatment, state only that practitioners should “advice people with an eating disorder who are exercising excessively to stop doing so”! [11]. While there is consensus amongst professionals that psychoeducation is a suitable approach for adolescents with compulsive exercise [12], no evidence-based therapeutic interventions have been designed specifically for young people with eating disorders.

The Loughborough Eating Disorder Activity Programme (LEAP) was the first evidence-based group intervention developed to address compulsive exercise for adults with eating disorders, using a framework of psychoeducation and Cognitive Behavioural Therapy (CBT) [10]. A randomised controlled trial [13] studied the efficacy of LEAP in conjunction with CBT, in comparison with CBT alone for outpatients. Both treatments significantly reduced compulsive exercise severity, and further analyses suggested that LEAP was more effective than CBT in reducing core aspects of compulsive exercise [14].

### Development of NEAT intervention (formerly junior LEAP)

Given the lack of evidence-based treatment for adolescent compulsive exercise, and the promising outcomes of LEAP, staff at Schoen Clinic Newbridge, a specialist hospital for eating disorders in adolescents, adapted the programme for a younger population.

Initially, the adult version of LEAP was used with adolescent patients at the hospital in a series of audits; however, the results showed that patients were unhappy in the programme and the attrition rates were high. As a result, LEAP was adapted to create NEAT (Newbridge eating-disorder activity treatment) (originally named JuniorLEAP [15] a brief intervention specifically for children and adolescents with eating disorder diagnoses. Adaptations included simplifying the psychoeducational content for young people, such as the explanation of the exercise maintenance formulation. PowerPoint slides were incorporated into the group sessions to allow the young people to access visual learning. The development was an iterative process involving patients and each version of NEAT was tested in audit before a version was found that held the children’s attention and interest.

An initial study explored the feasibility of NEAT for children and adolescents with eating disorders, by measuring psychopathology and compulsive exercise before and following completion of the group [15]. The study found the NEAT group therapy, alongside other therapies in a specialised inpatient setting, significantly reduced compulsive exercise as measured across all five subscales in the compulsive exercise test (CET) [16]. In addition, eating disorder psychopathology significantly reduced following completion of the group. Qualitative feedback suggested that participants found the sessions “helpful,” particularly in challenging misconceptions about exercise, learning strategies to reduce compulsivity, and understanding the compulsive exercise maintenance cycle. As the study did not compare the treatments efficacy to treatment as usual (TAU), the authors emphasised the need for further research to test NEAT treatment in a randomised control trial.

The present study using the definitive manualised version of NEAT has two primary aims.

- 1. To evaluate whether the NEAT group intervention reduces compulsive exercise in adolescents with an eating disorder diagnosis, when compared to TAU.
- 2. To explore whether the NEAT group reduces eating disorder psychopathology in adolescents with an eating disorder diagnosis, compared with TAU.

## Research design and methods

### Participants

Participants were inpatients receiving treatment at Schoen Clinic Newbridge (SCN), a specialised eating disorder hospital for children and adolescents. Inclusion criteria required meeting DSM-5 criteria for an eating disorder, while exclusion criteria included having previously completed NEAT or LEAP at the hospital, being below 75% Median BMI (%mBMI) being under 10 years, having a severe learning disability, being a day patient or having co-morbid psychosis.

Participants were randomly allocated to one of the two treatment groups: a ‘case’ group to complete the NEAT group alongside Treatment As Usual (NEAT + TAU) group or a ‘control’ TAU group.

A power calculation (available at request) was conducted based on the data from the initial study [15] and a sample size of *N* = 80 was agreed as sufficient to generate data. To prevent withholding an effective intervention from inpatients at Schoen Clinic Newbridge, it was further agreed that mean values would be examined at the mid-way point (*N* = 50). If the mean values are in line with our hypotheses at the mid-way point, data collection would stop and data analysis would begin.

### NEAT group

The NEAT intervention comprises 1 individual session to gain information around participants’ exercise habits, and 7 weekly group sessions which use a cognitive behavioural approach (see Table 1). NEAT is an active therapy group, which means that the patients take responsibility for behavioural change. Therapeutic homework of exercise monitoring records and a leisure activity questionnaire were provided for participants to complete between sessions. The therapists’ roles are to provide the information, guide the young people through the content, encourage and support to help them make the necessary changes. The therapy is manualised into a treatment protocol and was administered by Assistant Psychologists and a Psychotherapist employed at Schoen Clinic Newbridge receiving supervision from a Clinical Psychologist. The Manual and materials needed for the therapy are available in the Supplementary Materials, here. They were written by Gurdeep Aulakh and Amie Garghan with assistance from the other authors of this paper.

**Table 1:** NEAT programme outline

Session 0—NEAT exercise profile	Completion of NEAT exercise profile; completion of questionnaire pack
Session 1—Introduction to NEAT	Introduction to the NEAT group and its aims; definitions of keywords; introduction to maintenance formulation for compulsive exercise; introduction to monitoring records and leisure questionnaires
Session 2—Eating disorders and exercise	Introduction to Activity Anorexia Theory and weight and shape concerns; reflection on initiating and maintaining factors for exercise
Session 3—Exercise dependence	Introduction to positive and negative reinforcement; introduction to psychological dependence on exercise
Session 4—Compulsivity	Reflection on myths and facts about exercise; introduction to cognitive restructuring techniques; young people are challenged not to attend leisure activities, such as Yoga, which is on offer weekly
Session 5—Strict rules	Introduction to the relationship between holding beliefs about exercise and strict rules, resulting in behavioural rigidity. Young people are encouraged to consider own rules and encouraged to set more flexible and healthy ones
Session 6—Healthy exercise and relapse prevention	Reviewing the difference between healthy and unhealthy exercise behaviours; introduction of techniques to manage urges
Session 7—Review and reflection	Providing a space to reflect and review on NEAT. Consolidate knowledge and complete a risk management plan

### Treatment as usual

TAU refers to the standard inpatient programme at Schoen Clinic Newbridge. Treatment is provided by a multidisciplinary team, including dietitians, occupational therapists, nurses, psychologists, and psychiatrists. Patients may attend individual therapy, as well as group therapies providing psychoeducational content and addressing issues, such as body image and self-esteem. Patients may also be invited to groups to reintroduce physical activity, such as tai-chi, yoga, and other sports. Antidepressant and psychotropic medication may also be prescribed.

The treatment at Schoen Clinic Newbridge was rated ‘Outstanding’ by the Care Quality Commission (CQC) [22], The CQC is a UK governmental body which rates, inter alia, all British hospitals and ‘Outstanding’ is the highest rating. See Discussion for the possible implication of this.

### Measures

#### Compulsive exercise test (CET) [16]

The CET is a 24 item self-report scale measuring compulsive exercise. Participants rate how much they agree with a statement on a six-point scale from “Never True” to “Always True”. The CET measures five subscales of compulsive exercise: “Avoidance and Rule-Driven Behaviour”, “Weight Control Exercise”, “Mood Improvement”, “Lack of Exercise Enjoyment”, and “Exercise Rigidity”. A global score is calculated by totalling all subscales; a global score of ≤ 15 has been proposed as a cutoff to indicate compulsive exercise [17]. Internal consistency for the compulsive exercise test subscales, measured by McDonald’s omega, was good to excellent (0.80–0.95) in a study of female adolescents and adult inpatients to an eating disorder unit [18]. This measure was completed pre- and post- completed of the NEAT group (Time 1 and Time 2).

#### Eating disorder examination questionnaire (EDE-Q) [19]

The EDE-Q is 28 item self-report scale measuring eating disorder symptoms. Participants rate their agreement with statements on a seven-point scale ranging from “Not At All” to “Every Day” or “Markedly”. The EDE-Q measures four subscales of eating disorder psychopathology: “Dietary Restraint”, “Eating Concern”, “Shape Concern”, and “Weight Concern”. This measure was completed before and after the NEAT group (Time 1 and Time 2). Internal consistency measured by Cronbach’s alpha was excellent (0.96) in a study of adolescent females admitted to an inpatient unit for an eating disorder [20].

### Procedure

Patients with a %mBMI of at least 75%, were approached by an Assistant Psychologist to introduce the research study and seek consent for participation. For patients under the age of 16, informed parental consent and patient assent was sought, while for patients over 16, only informed patient consent was sought. Patients under 16 did not take part in the study unless both parental consent and patient assent had been received. Once consent had been obtained for ≥ 6 young people, randomisation into either TAU + NEAT or TAU groups took place using an Excel formula. After allocation, all participants completed Time 1 measures. After completion of the 7 weekly NEAT sessions, all participants completed Time 2 measures.

### Statistical analyses

Paired *t* tests were used to explore changes in compulsive exercise and eating disorder psychopathology following intervention.

A mixed analysis of variance test (ANOVA) was used to examine potential differences in self-reported compulsive exercise at week 1 and week 7 between the treatment groups. A secondary analysis using a mixed ANOVA explored the difference in self-reported eating disorder psychopathology between week 1 and week 7 for the two treatment groups.

The assumptions of normality were assessed using the Shapiro–Wilk test and homogeneity of variance was assessed using the Levene’s test for the equality of error variances.

## Results

### Participant characteristics

Child and adolescent inpatients (*n* = 91) were approached, and of these 75 (73 females, 2 males) consented to participate and were randomised to a treatment condition. The average age of the sample was 15.8 years (SD = 1.5), ranging from 12 to 17. Despite inclusion criteria accepting participants with any DSM-5 eating disorder diagnosis, all participants had a diagnosis of Anorexia Nervosa or Atypical Anorexia. Five of the sample had a co-morbid diagnosis of autism spectrum disorder (7%) and two had co-morbid obsessive–compulsive disorder (3%). As part of TAU, 32 received Psychotherapy (42%), 29 received CBT-E (38%), 10 received Art Therapy (13%), 1 underwent extended assessment and formulation work (1%), and 3 were not engaged in individual therapy (4%). 41 participants were randomised to the NEAT + TAU condition and 34 were allocated to TAU condition. Participants waited an average of 23 days between giving consent and starting the group rotation. Of this recruited sample, 24 (32%) did not complete the study. Reasons for non-completion included discharge prior to group completion (*n* = 9), consent withdrawn from research (*n* = 3), withdrawal from the NEAT group (*n* = 4), withdrawal due to non-appropriateness for the group (*n* = 1), and a group rotation being interrupted by a national lockdown due to COVID-19 (*n* = 7). As a result, 51 participants completed the study: 27 in the NEAT + TAU condition and 24 in the TAU condition. %mBMI was collected for these participants at both timepoints (see Table 2).

**Table 2:** %mBMI by experimental condition

Timepoint	NEAT + TAU (*n* = 27)		TAU (*n* = 24)	
	M	SD	M	SD
1	85.00	8.04	85.77	6.79
2	93.23	6.39	93.97	5.95

### Changes in self-reported compulsive exercise following intervention

Paired *t* tests were used to explore the differences in self-reported compulsive exercise following each intervention. Participants in both the NEAT + TAU and TAU interventions saw significant decreases in total compulsive exercise. The NEAT + TAU group saw significant reductions in all subscales except for ‘Mood Improvement’, while the TAU group saw significant reductions in most subscales, with the ‘Mood Improvement’ and ‘Lack of Exercise Enjoyment’ subscales showing no significant change (see Tables 3 and 4).

**Table 3:** Mean, standard deviation and change of total CET and CET subscales in NEAT + TAU group

	T1		T2		Pre- to post-group			
	Mean	SD	Mean	SD	Mean change	SD	*t*	*p*
Compulsive exercise (total CET)	16.42	4.51	12.92	4.83	3.50	4.44	4.10	< 0.001^b^
CET subscales								
Avoidance and rules	3.43	1.33	2.30	1.39	1.15	0.89	6.66	< 0.001^b^
Weight control	4.12	0.92	3.32	1.27	0.80	0.92	4.51	< 0.001^b^
Mood improvement	3.24	1.30	2.93	1.20	0.31	1.01	1.59	0.124
Lack of exercise enjoyment	2.48	1.57	1.91	1.40	0.57	1.13	2.61	0.015^a^
Exercise rigidity	3.59	1.26	2.46	1.60	1.13	1.15	5.12	< 0.001^b^

Mean change of exercise compulsion was identified through two-sided paired samples *t* tests

^a^ Statistical significance < 0.05

^b^Highly statistically significant < 0.01

**Table 4:** Mean, standard deviation and change of total CET and CET subscales in TAU group

	T1		T2		Pre- to post-group			
	Mean	SD	Mean	SD	Mean change	SD	t	*p*
Compulsive exercise (total CET)	14.86	3.95	12.17	4.85	2.68	2.94	4.47	< 0.001^b^
CET subscales								
Avoidance and rules	3.19	1.28	2.47	1.50	0.73	1.00	3.55	0.002^b^
Weight control	3.59	1.36	2.87	1.44	0.71	1.02	3.43	0.002^b^
Mood improvement	3.56	0.93	3.28	1.08	0.28	1.09	1.27	0.218
Lack of exercise enjoyment	1.26	1.07	1.29	0.97	− 0.03	0.81	− 0.17	0.868
Exercise rigidity	3.24	1.06	2.25	1.39	0.99	1.03	4.70	< 0.001^b^

Mean change of exercise compulsion was identified through two-sided paired samples *t* tests

^b^Highly statistically significant < 0.01

### Changes in compulsive exercise by treatment condition

A Shapiro–Wilk test indicated that assumptions of normality were violated; however, examination of the residuals using QQ plots found that there were no severe violations and the assumption of homogeneity of variance was met. A mixed ANOVA showed a significant difference in compulsive exercise at week 1 (*M* = 15.69, SD = 4.29) and compulsive exercise at week 7 (*M* = 12.57, SD = 4.80), regardless of condition (F(1, 49) = 33.54, *p* < 0.001, ηp2 = 0.41). However, there was no difference between the NEAT + TAU and TAU conditions in the degree to which compulsive exercise changes from week 1 to week 7 of intervention (F(1,49) = 0.99, *p* = 0.324, ηp2 = 0.02).

### Changes in self-reported eating disorder psychopathology following intervention

Paired *t* tests were used to explore the differences in self-reported eating disorder psychopathology following each intervention. Participants in both the NEAT + TAU and TAU interventions saw significant decreases in total eating disorder psychopathology and all subscales of the EDE-Q (see Tables 5 and 6).

**Table 5:** Mean, standard deviation and change of total EDE-Q and EDE-Q subscales in NEAT + TAU group

	T1		T2		Pre- to post-group			
	Mean	SD	Mean	SD	Mean change	SD	t	*p*
Eating disorder Psychopathology (total EDE-Q)	4.56	1.18	3.53	1.30	1.03	1.25	4.31	< 0.001^b^
EDE-Q subscales								
Dietary restriction	4.49	1.90	2.28	1.88	2.21	2.26	5.08	< 0.001^b^
Eating concern	3.58	1.32	2.85	1.36	0.73	1.04	3.65	0.001^b^
Weight concern	4.88	1.10	4.19	1.40	0.70	1.39	2.60	0.015^a^
Shape concern	5.27	0.93	4.82	1.41	0.45	1.14	2.06	0.049^a^

Mean change of eating disorder psychopathology was identified through two-sided paired samples *t* tests

^a^Statistical significance < 0.05

^b^Highly statistically significant < 0.01

**Table 6:** Mean, standard deviation and change of total EDE-Q and EDE-Q subscales in TAU group

	T1		T2		Pre-to-post-group			
	Mean	SD	Mean	SD	Mean change	SD	t	*p*
Eating disorder Psychopathology (total EDE-Q)	4.17	1.52	3.19	1.59	0.98	1.16	4.14	< 0.001^b^
EDE-Q subscales								
Dietary restriction	4.08	1.94	2.21	2.11	1.88	2.32	3.96	0.001^b^
Eating concern	3.25	1.48	2.42	1.53	0.83	1.16	3.51	0.002^b^
Weight concern	4.45	1.63	3.75	1.82	0.70	1.17	2.93	0.008^b^
Shape concern	4.89	1.55	4.38	1.63	0.51	0.99	2.51	0.019^a^

Mean change of eating disorder psychopathology was identified through two-sided paired samples *t* tests

^a^Statistical significance < 0.05

^b^Highly statistically significant < 0.01

### Changes in eating disorder psychopathology by treatment condition

A Shapiro–Wilk test indicated that eating disorder psychopathology was skewed; however, examination of the residuals using QQ plots found that there were no severe violations and the assumption of homogeneity of variance was met.A Mixed ANOVA showed a significant difference in eating disorder psychopathology at week 1 (*M* = 4.38, SD = 1.35) and compulsive exercise at week 7 (*M* = 3.37, SD = 1.44), regardless of condition (F(1, 49) = 35.33, *p* < 0.001, ηp2 = 0.42). However, there was no difference between the NEAT + TAU and TAU conditions in the degree to which eating disorder psychopathology changed between week 1 and week 7 of intervention (F(1,49) = 1.08, *p* = 0.304, ηp2 = 0.02).

## Discussion

To our knowledge, this is the first RCT to test a group intervention for compulsive exercise in eating disorders designed for a child and adolescent population. This study builds upon a previous evaluation of the efficacy and acceptability of NEAT [15], by comparing the intervention to treatment as usual in a specialised eating disorder inpatient hospital.

The literature suggests that LEAP-based interventions are effective in reducing compulsive exercise in anorexic populations [13, 15]. However, the present study found no differences between the NEAT intervention and TAU in reducing compulsive exercise, mirroring findings of studies of LEAP in inpatient [21] and outpatient [13] settings when comparing the intervention to standard treatment. However, new examinations of the data from Hay’s 2018 study [13], have shown some advantages of LEAP in reducing compulsive exercise compared to standard treatment [14].

The similar effects of a targeted compulsive exercise intervention and TAU in the present study, may reflect the efficacy of the ‘Outstanding’ treatment [22] provided at Schoen Clinic Newbridge, as rated by the Care Quality Commission (CQC), in which compulsive exercise were addressed with a multidisciplinary approach and interventions delivered by Psychiatrists, Psychotherapists, Occupational Therapists, Dieticians, and nursing staff. The CQC is a UK government body which monitors, inspects and rates all British hospitals. ‘Outstanding’ is the highest rating. It was given, because the clinic was committed to research and audit of all its therapies; its therapy innovation was praised, as was the coordination of clinical management resulting in high-quality care. It mentioned the staff were skilled and supported by training. It noted particularly that different professionals worked together effectively to assess and plan for a patient’s needs. The CQC also highlighted the clinic’s focus on safety and the well-maintained environment. This of course does raise the bar for any comparison.

The NEAT group was designed to be one component of a therapeutically dense inpatient environment. NEAT builds upon consensus that psychoeducation is an appropriate tool with which to address compulsive exercise [12] and it has been demonstrated that all aspects of the intervention (group sessions and therapeutic homework) were accepted by child and adolescent patients [15]. Acceptability of the intervention is further supported in the present study, as only a small proportion of attrition was due to withdrawal from the NEAT intervention. In outpatient settings which do not provide such a dense range of multidisciplinary interventions, we would argue NEAT could be used as a targeted intervention for children and adolescents for whom compulsive exercise is a primary concern. Anecdotal views of staff were that patients, usually those with severe issues with over-exercising, benefited more than others and we recommend that future research explores this further.

Our further aim was to examine the effects of intervention and TAU on eating disorder psychopathology. In line with the literature, [13, 15, 21] completing this specific compulsive exercise intervention saw reductions in psychopathology, but the effect was not significantly different from treatment as usual. It is, therefore, likely that the change in eating disorder psychopathology is explained by the multidisciplinary treatment provided at Schoen Clinic Newbridge.

## Limitations

A key limitation of the present study was the underpowered sample size. This study suffered 32% attrition, a common difficulty in studies of treatment for anorexia nervosa in which patients may attempt to retain symptoms, such as compulsive exercise [23]. It is important to note that most of the attrition was due to reasons external to the present study, such as patients’ being discharged from hospital or the disruption caused by the COVID-19 pandemic, and only a small number was due to disengagement with the NEAT intervention or the research process. The existing studies comparing LEAP to standard treatment have had similarly underpowered samples and have similarly found no differences between treatments. This indicates the need for larger scale testing of LEAP and NEAT to determine whether it is advantageous to TAU in treating compulsive exercise.

A limitation of the present study is that reintroduction to “healthy” exercise was uncontrolled. In an RCT [24], a group ‘Healthy Exercise Behaviour’ intervention which combined CBT and psychoeducation elements with built-in exercise sessions showed greater reductions of compulsive exercise than TAU. During the present RCT, some participants were participating in 1–3 h per week of activities (yoga, tai-chi, team sports) aimed to promote exercising for enjoyment, while others could not participate due to low weight or physical health risks. We cannot be sure whether NEAT with adjunctive exercise sessions could replicate the findings of Dittmer's study [24]

## What is already known on this subject?

Compulsive exercise is a weight-control behaviour that is linked to a more severe and longer course of illness in eating disorders. There is agreement as to the factors that would make up effective treatment (psychoeducation, cognitive behavioural framework); however, the NICE recommendation for treatment of compulsive exercise is limited and there is no widely endorsed group intervention. LEAP has shown promise as a group intervention for compulsive exercise in adults, while NEAT (previously JuniorLEAP) has shown promise in addressing compulsive exercise in children and adolescents with eating disorders.

## What does this study add?

Engagement with a group intervention adapted from LEAP to suit an inpatient child and adolescent population was related to reduced compulsive exercise and eating disorder symptoms, but to no greater effect than a specialised inpatient treatment for eating disorders delivered by a full MDT.

## Conclusion

Our findings show that while the NEAT group does not add value to TAU in a specialised inpatient setting, clinical researchers must always be mindful of the core Hippocratic principle of ‘do no harm’. No patient reported distress and no member of a large multi-disciplinary staff reported concerns with the therapy. It is the authors’ clinical belief that it may be an advantageous intervention for adolescents for whom compulsive exercise is a primary difficulty and, therefore, may be at greater risk of a severe course of illness. As such, further testing is required in larger populations, high compulsive exercise populations, and in outpatient settings. To facilitate this, the NEAT manual is available, here.

## Supplementary Information

Below is the link to the electronic supplementary material.

Supplementary Material 1.

## Data Availability

The therapist manual is attached to the electronic version of this paper. Reasonable access to raw data is available.
