# Supplementary material for: A randomised control trial study evaluating a compulsive exercise group for adolescent inpatients with eating disorders
Source: Eat Weight Disord. 2025 Dec 17;31(1):6. doi: 10.1007/s40519-025-01809-1 (PMC12830455; doi:10.1007/s40519-025-01809-1)
Supplement: Supplementary file 1 — Supplementary Material 1. [file 40519_2025_1809_MOESM1_ESM.pdf]

Aims

The primary aim of NEAT is to help children and adolescents gain control over their exercise NEAT promotes healthy attitudes, beliefs and behaviours towards exercise. NEAT does not advocate for the young people to stop exercising, it aims to equip individuals with knowledge and skills to assist them in regaining control.

- To educate young people on their relationship with compulsive exercise and how its maintained.
- To educate the young people on the possible factors that affect their attitudes, beliefs and behaviours towards exercise.
- To understand the difference between 'healthy' and 'unhealthy' exercise.
- To introduce cognitive skills to challenge attitudes and beliefs towards exercise.
- To introduce new coping strategies to manage exercise urges.
- To prepare for life outside of the group and risk management plan.

Structure

Session structure

All sessions apart from the Exercise Profile session are one hour long and facilitated by the Psychology department. Practitioner notes and tasks are aimed at fulfilling a therapeutic hour of 50 minutes, with 10 minutes flexibility. Each group can include 4-6 young people who are within a similar age range. NEAT is a closed group, therefore young people who decide to withdraw from the group at any stage are not re-invited to complete the group. Alternatively, they can recommence a new group if they wish to. This allows for the young people in the group to build confidence and trust in the practitioners and peers; it also encourages open discussions within the group.

Each session includes practitioner notes and PowerPoint presentations to support delivery of the programme. A handbook is also given to the young people which comprises of written tasks, key points from sessions and handouts. The young people can keep the handbook once they have completed the programme.

Each session also includes different tasks, some of which utilise laminated printouts (Appendix 2). It is important that these tasks are discussed as a group, the answers to each of these tasks has been inputted into tables within the practitioner notes and aids discussion.

Programme Summary

Individual session: NEAT Profile, 30 minutes

Completion of NEAT exercise profile and outcome measures. Discussion of suitability of group.

1. **Session 1: Introduction to NEAT**

   Introduction to the NEAT group and its aims; definition of key words; introduction to maintenance formulation for compulsive exercise; introduction to monitoring records and activity questionnaires.

2. **Session 2: Eating Disorders and Exercise**

   Introduction to Activity Anorexia Theory and weight and shape concerns; reflection on initiating and maintaining factors for exercise.

3. **Session 3: Exercise Dependence**

   Introduction to positive and negative reinforcement; introduction to psychological dependence on exercise.

4. **Session 4: Compulsivity**

   Reflection on myths and facts about exercise; introduction to cognitive restructuring techniques; young people are challenged not to attend a sports or physical activity for the week.

5. **Session 5: Strict Rules**

   Introduction to the relationship between holding beliefs about exercise and strict rules, resulting in behavioural rigidity. Young people are encouraged to consider own rules and encouraged to set more flexible and healthy ones.

6. **Session 6: Healthy Exercise and Urge Management**

   Reviewing the difference between healthy and unhealthy exercise behaviours; introduction of techniques to manage urges.

7. **Session 7: Review and Reflection**

   Provide a space to reflect and review on NEAT. Consolidate knowledge and complete a risk management plan

User Guide Information

The user guide is the complete set of practitioner notes that is required to facilitate each group session. The notes have been highlighted with keywords to assist with facilitation. Follow the instructions of each keyword until prompted by a different one. Please see below information for each keyword.

Task: Activity to be completed with/by the young person.

Share: Statements to be shared with the young person (this can be abbreviated or read directly from the notes).

Note: Information for the practitioner to consider.

Discuss: Points to be discussed with the young people.

Prompt: Answers to be shared or covered following discussions. The prompts assist in discussion points.

Homework Task: Homework for the young people to complete in their own time.

Accompanying the practitioner notes are PowerPoint slides for each session. The PowerPoint slides include summary points and images which are referred to in the practitioner notes. These slides have been created to provide interactive and visual learning for the young people. It may be helpful to review the practitioner notes and slides prior to facilitating a session.

At the beginning of each practitioner note is a resource section which details what you require for each session. The young person's handbook has been separated into sessions; therefore, the resource section refers to the handbook section required for each session.

Throughout the user guide there are multiple tasks, some of which utilise laminated printouts. There are tables which outline answers for many of the tasks; it is important that the answers in these tables are discussed and reviewed further as a group.

Exercise Profile

Resources

THIS SESSION IS COMPLETED 1:1

NEAT Handbook (Appendix 1.2), clipboard, pen / felt tips, calculator, EDEQ and CET questionnaires (Appendix 2.1 and 2.3).

INTRODUCTION TO NEAT (5 mins)

Share: What is NEAT?

- NEAT Cognitive Behavioural Therapy (CBT) group for compulsive exercise in Eating Disorders. (Briefly explain the CBT model – our thoughts, feelings and behaviours are all linked).
- The focus will be on the present and the future, and the factors that maintain your exercise behaviour.
- It will be a mix of slides, group discussions and tasks.
- NEAT is an active therapy group, which means that the young people will need to be able to take responsibility for behavior change. This means you will be asked to complete monitoring records to record your exercise urges through the week. You will also be asked to reflect on any activity you engage in through the week through an activity questionnaire. This is something we explore and reflect on at the start of each group session with support and boundaries
- Our role is to provide information, guidance, support and encouragement to support you to make changes. We will ensure that the group feels safe and set boundaries to provide you with a space to reflect on your relationship with exercise

Share: NEAT Aims

- To educate you on your relationship with compulsive exercise and what keeps it going/maintained.
- To educate you on possible factors that affect your attitudes, beliefs and behaviors towards exercise.
- To understand the difference between 'healthy' and 'unhealthy' exercise.
- To introduce cognitive skills to challenge attitudes and beliefs towards exercise.
- To introduce new coping strategies to manage exercise urges.
- To prepare for life outside of the group and risk management plan.

The overall aim is to help you gain CONTROL over your exercise and to have healthy attitudes, beliefs and behaviours towards exercise. It is not to stop you from exercising, but to equip you with knowledge and skills that will help you to regain control again.

COMPLETING THE EXERCISE PROFILE (20 mins)

Share:

The exercise profile helps you to understand reasons for exercising and the functions that exercise serves for you.

Task:

1. Ask the young person to work through each scale (pages 1-6) of the exercise profile in their handbook.
2. Once they have done this, calculate their scores by following the guidance in their handbook and shade in their graph (page 7) to provide a visual representation of their exercise profile.
3. Highlight the relevant explanations on the interpretation pages (page 8-10) and use these explanations to discuss what their exercise profile means.
4. Finally, discuss with the young person the type of exercise they typically engage in. Explore with the young person how their exercise relates with their profile.

COMPLETING QUESTIONNAIRES

Task:

Provide the young person with the EDEQ and CET questionnaires to complete. These can be scored at a later point.

SUMMARY AND CHECK OUT (5 mins)

Ask the young person whether they have any questions. Inform the young person of the date of their first session and check out.

Note:

Collect handbook at the end of each session. NEAT handbooks remain with the therapist until session 7.

Session One: Introduction to NEAT

Resources

Session 1 PowerPoint, NEAT Handbook (Appendix 1.3), pens, laminated formulation headings (Appendix 2.7), laminated formulation descriptions (Appendix 2.8), blue tac, whiteboard, whiteboard pens, NEAT Exercise Monitoring Records (Appendix 2.5) and NEAT Activity Questionnaire (Appendix 2.6).

INTRODUCTION TO NEAT (10 mins)

Discuss

Introduce names and engage in an open discussion about experiences of previous groups attended.

Share

This is what will be explored in each session:

1. Introduction to NEAT
2. Eating disorders and the link with exercise
3. Exercise Dependence
4. Compulsivity
5. Strict Rules
6. Healthy Exercise and Urge Management
7. Review and Reflection of NEAT

Discuss

Is there any content you feel might be useful to explore in the group or might find helpful?

Share

Give a brief outline of the what the group involves and revisit the aims from NEAT exercise profile session.

SETTING GROUP BOUNDARIES

Share

Group rules / boundaries are set to create a safe and comfortable group environment for everyone to speak openly about opinions, experiences and reflections if you want to.

Task

Ask each young person to set one rule and what would make the group more comfortable for them. Write these on a piece of paper.

Prompt

- Listening to others
- Confidentiality
- Respect others
- No exercising (including leg shaking)

Share

Two boundaries from the practitioners to help sessions run as smoothly as possible:

- Be prepared for sessions so they can begin on time.
- This is an educational group, you are here to learn and participate so you can take as much as possible from the group, there is no wrong answer.

Note

The group boundaries should be on display from the start of each session.

THE MAINTENANCE FORMULATION (10 mins)

Share

A formulation is a visual diagram that helps us to understand why a behaviour continues and breaks down the different factors that keep certain behaviours going. Knowing what factors maintain a behaviour helps us to target treatment.

Discuss

What might be some factors that maintain exercise behaviour?

Share

For exercise within eating disorders, the maintenance formulation includes factors such as dependence, compulsivity and strict rules. All these factors can contribute to exercise becoming compulsive. We will be defining compulsive exercise later in the session.

Task

Using the PowerPoint slides introduce the young people to the maintenance model below.

Share

The formulation may not make much sense at the moment, but each session will focus on a different section of the maintenance formulation. By the end of the programme you should have a good understanding of how your exercise behaviour started, how it may have become compulsive and what makes it difficult to stop. At the beginning of each session you will be asked to complete the formulation.

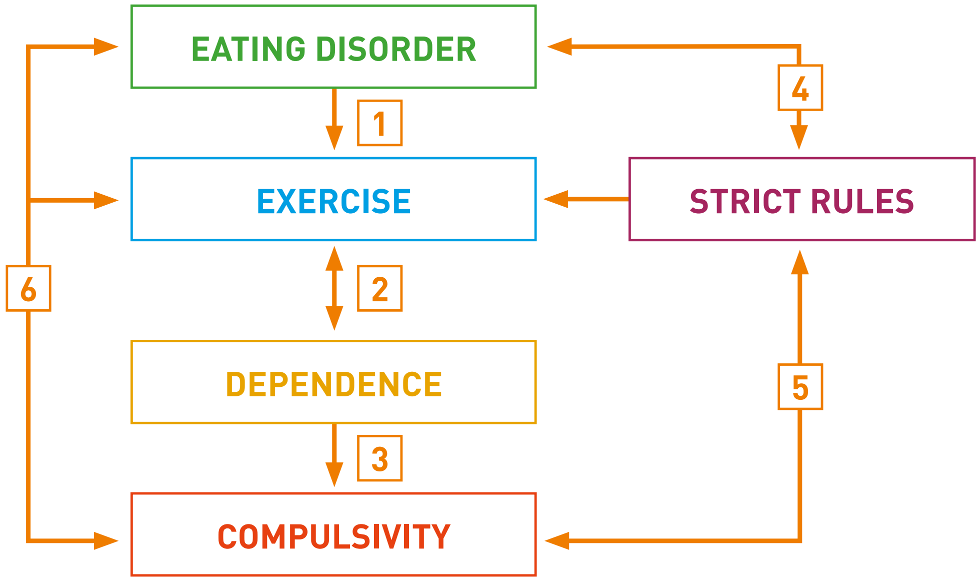

Task

Using the laminated formulation headings stick the cut-outs to a board using blue tac and draw out the diagram. Provide each young person with a laminated formulation description card and ask the young people to see if they can match the descriptions to the different relationships in the maintenance formulation. Below are the statements which match the diagram above.

Discuss

Discuss each statement with the young people and ensure they have a good understanding of the formulation.

1. Having an eating disorder can make someone more likely to engage in exercise behaviour / activity. This is because of weight and shape concerns and an increased urge to be active at a low weight.
2. When someone begins to engage in exercise behaviour, they may feel that it improves their mood or prevents them from feeling negative emotions, which increases the risk of dependence on the exercise behaviour.
3. Beliefs and fears about the negative impact of stopping exercise means that the exercise behaviour can become compulsive (difficult to stop).
4. When someone is concerned about controlling their shape and weight, they may set themselves very strict rules around their exercise behaviour, which again, makes it difficult to stop or adapt these. Many people with eating disorders experience some level of perfectionism and are rigid in their thinking, which means these rules are often followed perfectly.
5. Following these strict rules can lead to exercise behaviour becoming compulsive (difficult to stop).
6. When someone is concerned about their weight and shape, they may have beliefs and fears about the impact on weight if they stop exercising. This too can lead to the exercise behaviour becoming compulsive (difficult to stop).

EXERCISE (15 mins)

Share

We are going to be using a lot of terms over the next 6 weeks, so it important that you understand what these terms mean.

Discuss

What is the difference between 'physical activity' and 'exercise'?

Share

Physical activity refers to any bodily movement that uses the muscles in the body. Exercise is a physical activity that is planned or structured with the primary aim of influencing weight and shape or any component of physical fitness (muscular strength or endurance, flexibility, the ability for your heart and lungs to use oxygen for extended periods of physical activity, improved heart rate).

Task

Ask the young people to refer to their handbooks and write down the terms.

Ask the young people to work together as a group to create a brainstorm of types of exercise and physical activity.

Prompt

Physical activity: Lifting your arms in the air, walking, scratching your head, fidgeting with your fingers.

Exercise: Running, sport specific training (football, netball, hockey etc.), fitness classes, swimming, weight lifting.

Share

There is a type of exercise, called incidental exercise, which refers to physical activity that is not planned or structured, but still has a secondary effect of influencing weight, shape, or physical fitness.

Discuss

Can you think of any examples of incidental exercise?

Prompt

Vigorous housework / gardening, long or regular dog walks, walking to school every morning, fidgeting or just being generally active.

Share

For this reason, it is important that the you remember to think of exercise as including all forms of physical activity; such as tidying your room, walking around the shops or being restless. Incidental exercise needs to be considered alongside the planned and structured exercise. All physical activity, whether this is exercise or not, can have positive health benefits, but can also have a negative effect if this is compulsive.

WHAT DO WE MEAN BY COMPULSIVE? (5 mins)

Share

Compulsive does not refer to quantity.

Compulsive means:

- Any activity that is motivated by disordered eating attitudes or beliefs.
- Any activity that can have a negative effect on either physical or psychological health.
- Any activity that cannot be easily stopped or altered.

Activity is compulsive if it is any of the above, regardless of how much or how often it is done.

Discuss

How would we link this to exercise?

Task

Underneath the young people's definition of exercise, ask them to now write a definition for 'compulsive exercise' using the information they now have about what compulsive means.

Share

Compulsive exercise refers to physical activity that is motivated by disordered eating attitudes and beliefs that has a negative impact on either physical or psychological health. It is characterised by an inability or unwillingness to cut down or stop exercising despite this negative impact.

MONITORING RECORDS AND ACTIVITY QUESTIONNAIRE (10 mins)

Monitoring Records

Share

As part of the active process of NEAT, we will be asking you to complete monitoring records between each session, to monitor your exercise urges.

- Provide the young people with a copy of the monitoring records which outlines the information below. Briefly explain these. Go through the example monitoring record on the slides to show the young people how to complete them.

Share

Monitoring is a good way of assessing 'in-the-moment' thoughts, feelings and behaviours. It involves recording urges in real time (as they happen / in-the-moment).

"Monitoring helps people to change. By becoming aware of what you are doing, thinking and feeling at the very time that it is happening, you learn that you have choice, and that things that you thought were automatic, or out of your control can be changed with attention, effort and practise." (C.Fairburn, 2008)

If we relate this to exercise, it will enable you to be more aware of your exercise urges and therefore manage them in much more healthy way.

Monitoring records will be reviewed weekly, at the beginning of each session, to see how you have managed your exercise urges over the week.

Discuss

How do you feel about sharing your monitoring records within group sessions?

Note

Reach an agreement with the young people that will encourage them to share with the group (ie: if one person shares everyone shares their records, anything that is too difficult to share does not have to be disclosed). Reassure the young people feelings may arise from sharing records, this will be addressed with the group and its likely their peers will relate to how they feel.

Share

Urges recorded may be shared with wider teams, if felt necessary so that support can be offered around difficult times. Reassure the young people that their team will be aware that they are working on their urges through attending the group.

Activity Questionnaires

Share

In addition to the monitoring records, we will be asking you to complete activity questionnaires between each session, to explore urges that occur for you whilst engaging in activity.

- Provide the young people with a copy of the activity questionnaire and explore the questionnaire with them.

SUMMARY (5 mins)

Discuss

Any questions?

- What are the key things that you will be taking away from today's session?

Share

What are the key things that you will be taking away from today?

- We will be working together to help increase knowledge around healthy and unhealthy exercise, with you taking a lead role in regaining control of your exercise behaviour.
- Exercise is any physical activity that is planned and structured, with the aim of influencing weight, shape or physical fitness.
- Exercise can have positive health benefits but can also have a negative effect if this becomes compulsive.
- Compulsivity is not related to quantity.

Session Two: Eating Disorders and Exercise

Resources

Session 2 PowerPoint, NEAT Handbook (Appendix 1.4), pens, laminated formulation headings (Appendix 2.7), blue tac, whiteboard, whiteboard pens, Activity Anorexia Quiz (Appendix 2.9), NEAT Exercise Monitoring Records and NEAT Activity Questionnaire (Appendix 2.5 and 2.6)

REVIEW MONITORING RECORDS AND ACTIVITY QUESTIONNAIRES (5 mins)

Discuss

Go around each person in the group and ask them to share and reflect upon what they recorded in their monitoring records and activity questionnaires from Session 1:

- How have they found filling them out?
- Did they manage to resist any exercise urges?
- What did they notice, (ie; what worked to resist an urge, were there any similar themes that led to an urge?)
- If not completed, what are the reasons for this? Discuss ways in which the young people could overcome these obstacles.

RECAP FORMULATION AND INTRODUCTION TO SESSION TWO (5 mins)

Task

Using a board and the laminated NEAT Formulation headings, ask the young people to get up and recreate the maintenance formulation for exercise. They can use blue tac to stick these up and the whiteboard pens to draw the relevant arrows.

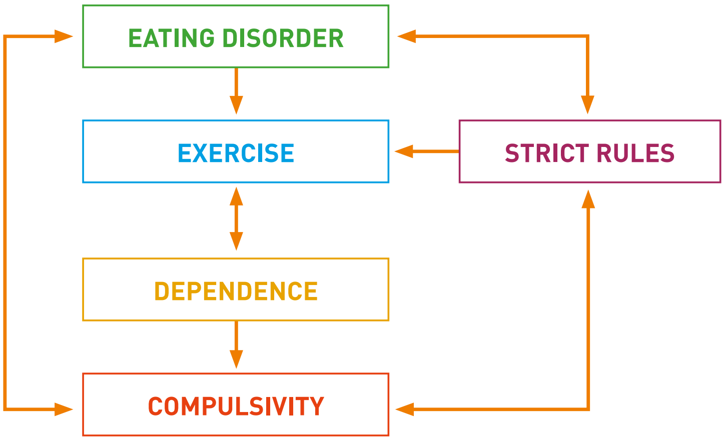

INTRODUCTION

Introduce session two and read out the aims.

- Session two will look at the 'Eating Disorder' section of the maintenance formulation.

By the end of session two you should know:

- How exercise behaviour may start in the first instance, specifically in people with eating disorders.
- An understanding of the activity anorexia theory.

WHY DO PEOPLE EXERCISE? (10 Mins)

Discuss

Why do people exercise?

Prompt

Because its 'healthy', to keep fit, to improve mood, it's enjoyable, to socialise, for achievement, as a hobby, to compete.

Share

The reason someone may start exercising is often not the same as the reason that someone cannot or doesn't want to stop. Exercise is in fact a healthy, life improving activity that you should enjoy. Reasons for beginning to exercise are known as 'initiating factors', and reasons for being unable to stop are known as 'maintaining factors'.

Task

Ask the young people to read through and complete the 'Initiating and Maintaining Factors' task on pages 15-17 in their handbooks to identify their own. Highlight this task will help them have a greater understanding of the functions of their own exercise, and therefore the most relevant sections of the maintenance formulation as we work through treatment.

Discuss

Has your exercise behaviour changed since the onset of your eating disorder? How has this changed? What were your motivations to begin exercising in the first place?

EATING DISORDERS AND EXERCISE (20 mins)

Discuss

Why might someone with an eating disorder be more likely to exercise?

Share

Weight and shape concerns and their control are at the heart of eating disorder difficulties. Many people with eating disorders engage in weight control behaviours such as food restriction. It may be that for some people, exercise is purely for weight and shape control. The exercise behaviour therefore came after their weight and shape concerns as part of the eating disorder. For others, healthy exercise may have been 'hijacked' by the eating disorder as another form of controlling shape and weight.

- The young people can refer to scale 5 of their exercise profile (see page 6): weight and shape exercise as an indicator of this.

Task

Another way of assessing the relationship between the eating disorder and exercise behaviour is to measure whether the young people ever experience exercise attitudes, beliefs and behaviours that are influenced by the eating disorder. Ask the young people to complete the short questionnaire on page 18 in their handbooks. Review this task and get the young people to reflect on their score.

ACTIVITY ANOREXIA

Share

In an experiment into the effect of food restriction on activity levels, two groups of rats were given access to running wheels.

Group One: Given enough food to maintain their 'normal' bodyweight.

Group Two: Food was restricted so that the rats began to lose weight.

Discuss

What do you think happened to the exercise levels of each group of rats? Did it stay the same, increase or decrease?

Share

Group One: Exercise levels remained the same.

Group Two: Exercise levels increased, e.g. spending longer and longer on the wheel, until they ran themselves to exhaustion, and in some cases death.

The activity anorexia model helps to demonstrate an urge to be active in individuals who are underweight.

- The young people can note down their answers on page 19 of their handbook.

THEORIES EXPLAINING ACTIVITY ANOREXIA

Biological Theory

Share

The urge to be active is controlled by a brain chemical called Leptin. In the rat experiment, the rats with a low body weight (the group who had their food restricted) had a significantly reduced level of Leptin. Therefore, they had no signal to stop exercising. Leptin levels increased as they restored to a normal bodyweight, their activity levels began to normalise again.

Increasing the amount of food given to the rats before they were given the opportunity to exercise did not affect the rats Leptin levels, suggesting that it is the low weight, rather than the restricted diet that causes Leptin levels to decrease. There is a graph on page 19, which represents Leptin levels returning to baseline as BMI returns within a normal range.

Evolutionary Theory

Share

The 'urge' to be active and the increase in energy levels is an evolutionary survival mechanism. The body responds to a fall in body weight by increasing energy levels so that animals can cover a wider area of land to find food. When the body is receiving enough food, and body weight begins to return to normal, energy levels should reduce.

- The young people can note down their answers on page 20 of their handbook.

Discuss

Can anyone relate to the feeling of restlessness / difficulty relaxing at a low body weight?

Share

Increase in energy can sometimes manifest into fidgeting or incidental exercise, such as walking around more than usual. Not all individuals experience an increased 'urge' to exercise. The increase in energy and activity is only meant to be short term survival mechanism. It places considerable stress on the body to release such a large amount of energy, and the increased activity can be damaging to the body. It is important that activity levels are controlled so that health is not damaged.

Task

Ask the young people to hand in their handbook and hand out the Activity Anorexia quiz. Ask the young people to complete the quiz to test how much they have learnt about the activity anorexia theory.

SUMMARY (5-10 mins)

Discuss

Any Questions?

- What are the key things that you will be taking away from today?

Share

- The reason for starting to exercise may not always be the reason that exercise behaviour continues.
- An eating disorder can lead to someone beginning to engage in exercise behaviour because of weight and shape concerns.
- An eating disorder can lead to someone beginning to engage in exercise behaviour because of biological and evolutionary survival mechanisms.

Give the young people blank Monitoring Record and Activity Questionnaire.

Session Three: Exercise Dependence

Resources

Session 3 PowerPoint, NEAT Handbook (Appendix 1.5), pens, laminated formulation headings (Appendix 2.7), blue tac, whiteboard, whiteboard pens, laminated examples of positive and negative reinforcement (Appendix 2.10), NEAT Exercise Monitoring Records and NEAT Activity Questionnaire (Appendix 2.5 and 2.6).

REVIEW MONITORING RECORDS AND ACTIVITY QUESTIONNAIRES (5 mins)

Discuss

Go around each person in the group and ask them to share and reflect upon what they recorded in their monitoring records and activity questionnaires from Session 2:

- How have they found filling them out?
- Did they manage to resist any exercise urges?
- What did they notice, (ie; what worked to resist an urge, were there any similar themes that led to an urge?)
- If not completed, what are the reasons for this? Discuss ways in which the young people could overcome these obstacles.

RECAP FORMULATION AND INTRODUCTION TO SESSION THREE (5 mins)

Task

Using a board and the laminated NEAT Formulation headings, ask the young people to get up and recreate the maintenance formulation for exercise. They can use blue tac to stick these up and the whiteboard pens to draw the relevant arrows.

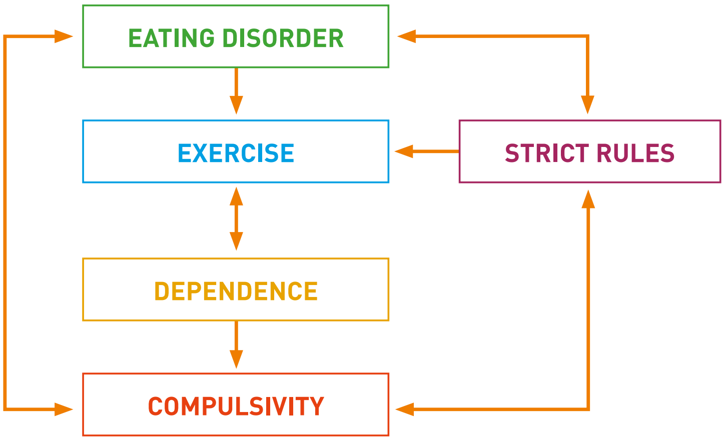

INTRODUCTION

Introduce session three and read out the aims.

- Session three will look at the 'Dependence' section of the maintenance formulation.

By the end of session three you should know:

- How exercise dependence is related to our mood.
- The difference between positive and negative reinforcement, particularly in relation to exercise.
- How the treatment of compulsive exercise is similar to the treatment of addictions.

EXERCISE DEPENDENCE (15 mins)

Share

Dependence on exercise is related to 'mood regulation'. Mood regulation refers to the ability to manage and respond to emotional experiences. If a person has difficulty managing their mood, then they may rely on exercise to do this. It is well known that exercise can have a powerful effect on mood.

Discuss

Has anyone ever heard of positive reinforcement and negative reinforcement?

Prompt

Positive Reinforcement: An action that results in something being added, such as a positive outcome or reward.

Negative Reinforcement: An action that results in something being taken away, usually something undesirable.

Both increase the chances of the behaviour being repeated.

Task

Handout the laminated Positive and Negative Reinforcement statements to each young person. Ask the young people to read the statements and separate them into two columns.

Note

It is important to highlight that reinforcement is about increasing the chances of a behaviour being repeated through a positive outcome or the removal of a negative outcome / feeling

Table 1: Answers for Positive and Negative Reinforcement Statements

Positive Reinforcement	Negative Reinforcement
**Ben is allowed to watch television once his homework is completed** – Ben gets something positive out of completing his homework, and the quicker he completes it the quicker he can gain his reward. This makes it more likely that Ben will complete his homework quickly in the future.	**Tim left his shoes in the middle of his bedroom and tripped over them so he tidies them away in his wardrobe** – Tim is more likely to keep his room tidy and put his shoes away properly to avoid tripping over.
**Susan gives her daughter £5 for every chore that she completes** – Susan's daughter is more likely to complete chores in the future.	**Claire feels stressed after a hard day at work, she engages in some meditation** – Claire is likely to find that meditating makes her feel less stressed, she is therefore more likely to meditate after work to relax herself.
**Spot the dog is given a treat after he sits when commanded to by his owner** – spot learns that if he sits he will get a treat, therefore he is more likely to sit when told in the future.	**Maggie is at the beach and can feel her shoulders burning, she puts on some sun cream** – By putting on sun cream, Maggie is removing the negative feeling of burning and avoiding sun burn. This makes it more likely that Maggie will put sun cream on in the future.
**Emily earns a star on her chart for every good deed that she performs in the classroom** – Emily is more likely to perform good deeds in the future.	**John feels cold while at a football match, so he puts on his coat** – Once he puts his coat on, John will no longer feel cold. This makes it more likely that John will take or wear a coat to football matches in the future.

Discuss

How does this relate to exercise?

Share

Positive reinforcement: Someone may exercise because it makes them feel good physically or psychologically, as exercise leads to biochemical changes in the brain. Here, positive emotions are added from exercise.

Negative reinforcement: Someone may exercise to avoid feeling negative moods such as anxiety or depression (negative reinforcement). Here, negative emotions are taken away from exercise.

Even a small amount of exercise (as little as 20 minutes of mild or moderate physical activity) has been shown to improve most people's mood and make them feel less anxious or depressed.

Note

Relate this to the young people's initiating and maintaining factors worksheet on page 15-17.

Share

Someone may have started to exercise because they wanted to join a club for social benefits, however, once they started to exercise, they found it made them feel good and improved their mood.

Discuss

Did anyone find that exercise improved their mood or made them feel good?

Note

Scales 1 and 2 on the exercise profile (page 8) relate to mood regulation: the higher the scores, the more significant this factor is in maintaining the exercise behaviour.

Task

Ask the young people to read through and rate the first scale on page 22 in their handbook. They will be working through a number of the scales throughout the session, the questions will help the young people gain a greater understanding of the mood regulating effects and their dependence on exercise behaviour.

EXERCISE 'ADDICTION' (5 mins)

Share

The powerful effect on mood can, over time, lead to a psychological dependence on exercise, which refers to a strong desire to continue the behaviour. This experience is sometimes referred to as an 'addiction'.

It is not possible to become physically addicted to exercise, however, the psychological dependence can feel so strong that it feels identical to a real addiction. During the NEAT programme, we will refer to the need to continue exercising as 'dependence' rather than 'addiction'.

It is important to note that many people experience the positive effects of exercise but would not describe themselves as dependent. It is only when exercise is needed to feel good, that the individual may feel dependent on exercise.

Task

Ask the young people to read through and rate the second and third scale on page 22 in their handbooks, which refers to their exercise dependence (addiction).

EMOTIONAL WITHDRAWAL SYMPTOMS (5 mins)

Share

When someone cannot exercise, they may experience negative or unpleasant mood changes, which is sometimes referred to as emotional withdrawal symptoms. Such as anxiety, anger, irritability, low in mood, frustration. This makes it extremely difficult to stop or cut down exercise behaviour, and exercise continues as a way of avoiding feeling these negative emotions (negative reinforcement).

Feeling good after you exercise (positive reinforcement) is a very powerful maintaining factor for exercise, however, exercising to avoid feeling negative mood states (negative reinforcement) is much more powerful, and is more common in compulsive exercise.

Discuss

How would/do you feel when your exercise is restricted?

Task

Ask the young people to read through and rate the fourth and fifth scale on page 23 in their handbooks, which refers to their emotional withdrawal symptoms if they cannot exercise.

EXERCISE RESTRICTION (10 mins)

Discuss

Why might it be important to restrict or manage activity level when you are a low weight?

Prompt

- It is important to restore weight and reach a minimum healthy weight so that the body functions at its best. Exercise can impact this and can lead to weight loss, therefore slowing the process of restoring weight.
- Health implications of exercising at a low weight: many young people are significantly dehydrated; exercising can worsen this. When someone exercises at a low weight, the body will start to break down muscle, this could include organ tissue such as the heart.
- Your body needs a lot of energy to repair. Introducing activity needs to be matched with dietary intake to ensure the body has enough energy.

Task

Ask the young people to write down why it is important to reduce activity when at a low weight in their workbooks (page 23).

Treatment For Other Addictions

Discuss

As exercise dependence can feel like a real addiction it's important to explore treatment to other addictions. How are other addictions treated?

Prompt

- Period of complete abstinence.
- Learning new, healthier coping strategies.
- Remodeling healthy behaviour.
- Monitoring records are useful to identify triggers.
- Require a gentle / graded reintroduction to keep it under control.

Treatment for other addictions is all about regaining control of the behaviour. It is important that exercise dependence is treated similarly to forms of addiction.

SUMMARY (5-10 mins)

Discuss

Any questions?

- What are the key things that you will be taking away from today's session?

Share

- Dependence on exercise is associated with its impact on our mood.
- Exercise can make us feel good (positive reinforcement) or prevent us from feeling bad (negative reinforcement), or sometimes both.
- The experience of exercise dependence is similar to other physical addictions, and therefore needs to be treated in the same way – by stopping the behaviour and regaining control.

Give the young people blank Monitoring Record and Activity Questionnaire.

Homework Task

Set the young people a challenge of choosing something enjoyable and less active to do this week instead of participating in a weekly activity. This challenge will be reviewed at the start of the next session.

Session Four: Compulsivity

Resources

Session 4 PowerPoint, NEAT Handbook (Appendix 1.6), pens, laminated formulation headings (Appendix 2.7), blue tac, whiteboard, whiteboard pens, laminated myths and facts statements (Appendix 2.11), NEAT Exercise Monitoring Records and NEAT Activity Questionnaire (Appendix 2.5 and 2.6).

REVIEW MONITORING RECORDS AND ACTIVITY QUESTIONNAIRES (5 mins)

Discuss

Go around each person in the group and ask them to share and reflect upon what they recorded in their monitoring records and activity questionnaires from Session 3:

- How have they found filling them out?
- Did they manage to resist any exercise urges?
- What did they notice, (ie; what worked to resist an urge, were there any similar themes that led to an urge?)
- If not completed, what are the reasons for this? Discuss ways in which the young people could overcome these obstacles.

Review activity challenge set in session 3: Did they complete the challenge to not attend a physical activity this week?

RECAP FORMULATION AND INTRODUCTION TO SESSION FOUR (5 mins)

Task

Using a board and the laminated NEAT Formulation headings, ask the young people to get up and recreate the maintenance formulation for exercise. They can use the blue tac to stick these up and the whiteboard pens to draw the relevant arrows.

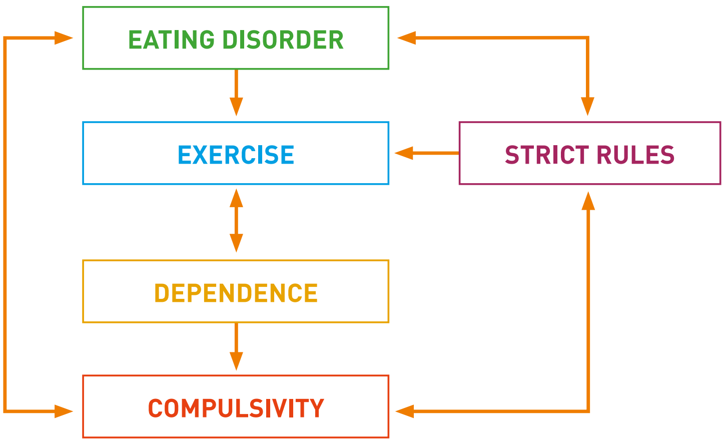

INTRODUCTION

Introduce session four and read out the aims.

- Session four will look at the 'Compulsivity' section of the maintenance formulation.

By the end of session four you should know:

- How our beliefs can lead to compulsive exercise.
- The difference between a myth and a fact.
- Some of the beliefs and assumptions that you hold that may be maintaining your exercise behaviour.
- Whether your beliefs are based on a myth or a fact.

WHAT ARE MYTHS AND FACTS? (20 Mins)

Share

Compulsivity does not refer to amount or frequency. If a behaviour has a negative impact on health regardless of how much or how often it is done, and it cannot be easily stopped or changed then it is considered compulsive.

Fears about what will happen if we stop exercising can often be based on myths that we think are true. These myths can therefore contribute to the maintenance of exercise because they reinforce the irrational belief. For example, "If I don't exercise, my muscle will turn to fat".

However, not all beliefs are based on solid facts, it is important to have a good understanding of the difference between a myth and a fact and identify whether our beliefs have any evidence to support them.

Discuss

What is the difference between a myth and a fact?

Prompt

Myth: A popular belief that is false or unsupported by evidence.

Fact: Something that can be supported by evidence.

Share

Lots of common myths have arisen from trying to make sense of relationships that have been observed. For example, it has been observed that you are more likely to catch a cold in the winter. The myth is that cold temperatures lead to catching a cold. Myths are usually expressed as an assumption in the form of "IF…. THEN…" statements. In this example "if I do not wrap up warm, then I will catch a cold".

Discuss

Has anyone ever heard of this statement? Do you agree that it is a myth?

Share

There is no real evidence that this is true. Evidence suggests that to catch a cold, you have to be exposed to someone who has a cold, and it is therefore more likely that we catch colds in the winter because we spend more time indoors due to the cold weather outside.

Many assumptions can serve us well, or in the example or wrapping up warm, at least do no harm. "If I study hard, then I will get top marks" is another example of an assumption that serves us well. There is some evidence to suggest that extra work will lead to better marks, but the assumption is not based on or supported by real facts.

However, other assumptions can have a negative impact on health, such as "If I do more exercise, then I will be healthier, fitter and faster". This could lead to over training, and therefore lead to being unhealthy, less fit and slower. Evidence tells us that there is only so much exercise the body can take before the positive effects are outweighed by the negative effects.

Task

Handout the myths and facts laminated statements to each young person. Ask them to read out and decide whether each statement is a 'myth' or a 'fact' and put these into columns. Once they feel they have completed the task, go through the answers, and ask them to tick these off on page 24.

Table 2: Answers for Myth and Fact Statements and Further Explanation.

Myths	Explanation
All body fat is unhealthy.	Only excess body-fat is unhealthy, which is why healthy weight is the target weight.<br>Body-fat is essential for the healthy functioning of the body (includes brain, heart etc.) and protects the internal organs.<br>It is the body's insulation against heat loss.
Walking to the shops or train station is not exercise.	This is incidental exercise and day to day movement.<br>It uses energy but isn't a planned form of exercise.
You need to be thin to be healthy.	What is thin? This is a subjective description.<br>We should consider weight based on being underweight, overweight or healthy weight. Someone who is underweight would not be healthy, therefore thinness does not mean improved health.
Fat can be turned into muscle.	Fat and muscle are two completely different types of tissue, just as your heart and lungs are different. Just because they may be in the same part of your body doesn't mean one can become the other.<br>The relationship between these two tissue types is complex.<br>**TASK:** As a group, read through the The Body Builder Analogy on page 25 which explains this further.
If you are fit, then you are healthy.	Many athletes over train, this is not healthy despite being fit.<br>Health refers to physical and mental health. Being fit only refers to physical health
You need to be thin to be fit.	Swimmers, rugby players etc. are all very fit despite often being essentially overweight according to BMI charts.
The human body is naturally thin.	If we apply this to babies, a thin baby is seen to be unhealthy. We already know our bodies need fat.
If a muscle is not used, then it turns to fat.	Muscle can lose tone if not used but does not become another type of tissue. It simply gets broken down by the body.
You cannot be fat and fit.	Fat is a subjective description. Our bodies need fat to function, but you can be overweight and still be physically fit (such as rugby players etc).

Facts
It takes less exercise to maintain fitness than it does to improve it.
Exercise can change/regulate your mood.
Exercise can change/regulate your mood.
Muscle weighs approximately three times as much as fat.
The thinner you are the more obsessional and rigid you become.
Exercise can be addictive.

PERSONAL BELIEFS AND ASSUMPTIONS (15 mins)

Task

Ask the young people to think about their own beliefs regarding exercise, by completing the questions on page 27 in their handbook. (They can look back at their fears about stopping exercise on page 17 to help identify their beliefs/assumptions).

Ask the young people to complete the cognitive restructuring sheet on page 28. An example of a completed sheet is available on the slides.

Discuss

What did you notice when exploring your belief? Encourage the young people to use this template to explore other unhelpful beliefs.

SUMMARY (5-10 mins)

Discuss

Any questions?

- What are the key things that you will be taking away from today's session?

Share

- A myth is a popular belief, whereas a fact is something that can be supported by evidence.
- Lots of myths arise from trying to understand the relationship between two things, for example, an increase in colds during the winter.
- Fears about what will happen if we stop exercising can often be based on myths that we assume are true.
- These fears / irrational beliefs can lead to exercise becoming compulsive, because they make it difficult to stop or change the behaviour.
- Looking at evidence for and against your beliefs can help identify if it is a myth or a fact.

Give the young people blank Monitoring Record and Activity Questionnaire.

Session Five: Strict Rules

Resources

Session 5 PowerPoint, NEAT Handbook (Appendix 1.7), pens, laminated formulation headings (Appendix 2.7), blue tac, whiteboard, whiteboard pens, laminated healthy and unhealthy rules (Appendix 2.12), NEAT Exercise Monitoring Records and NEAT Activity Questionnaire (Appendix 2.5 and 2.6).

REVIEW MONITORING RECORDS AND ACTIVITY QUESTIONNAIRES (5 mins)

Discuss

Go around each person in the group and ask them to share and reflect upon what they recorded in their monitoring records and activity questionnaires from Session 4:

- How have they found filling them out?
- Did they manage to resist any exercise urges?
- What did they notice, (ie; what worked to resist an urge, were there any similar themes that led to an urge?)
- If not completed, what are the reasons for this? Discuss ways in which the young people could overcome these obstacles.

RECAP FORMULATION AND INTRODUCTION TO SESSION FIVE (5 mins)

Task

Using a board and the laminated NEAT Formulation headings, ask the young people to get up and recreate the maintenance formulation for exercise. They can use the blue tac to stick these up and whiteboard pens to draw the relevant arrows.

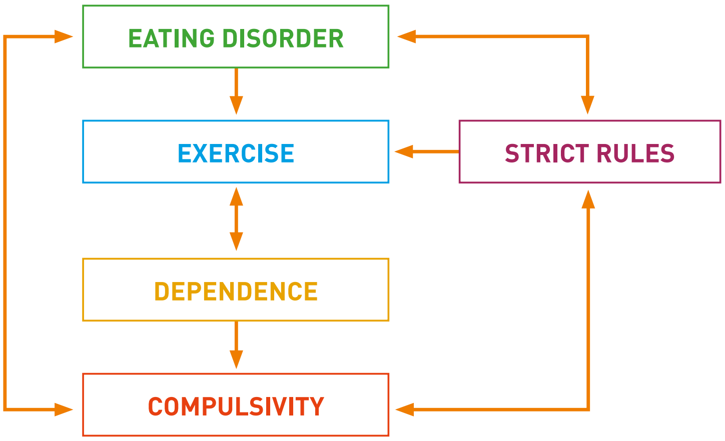

INTRODUCTION

Introduce session five and read out the aims.

- Session five will look at the 'Strict Rules' section of the maintenance formulation.

By the end of session five you should know:

- An understanding of behavioural rigidity.
- The relationship between the eating disorder, behavioural rigidity and compulsive exercise.
- The difference between healthy and unhealthy rules for exercise.
- How to set more flexible, healthy rules.

WHAT ARE STRICT RULES (5 mins)

Share

Having strong beliefs about what will happen if you stop exercising can lead to the creation of strict rules. For example, a belief of "if I stop exercising my muscle will turn to fat" may lead to a strict rule about lifting weights each morning before school.

Strict rules include following specific routines or order of events, and experiencing feelings of anxiety, irritability, or anger when a routine is disrupted or broken. Many people set rules for living, to support them in day-to-day life, however, strict and inflexible rules can lead to compulsive behaviours which are not always healthy.

Discuss

Can you think of any examples of strict rules, these can be general ones?

Prompt

I must always brush my teeth in the morning, I must always arrive to work 5 minutes early, I should always take the stairs instead of the lift, I must always wash my hands after going to the toilet.

BEHAVIOURAL RIGIDITY (5 mins)

Share

Behavioural rigidity refers to being inflexible (having to do things a certain way or in a certain order). It is not unique to exercise, and people who have behavioural rigidity tend to apply this to many areas of their life.

In relation to exercise, it is one of the most powerful maintaining factors for compulsive exercise. This is for 2 reasons:

1. The rigidity around exercise may have started because of a strong belief about what will happen if the exercise is stopped. In the previous example, it would be very difficult to stop doing weights in the morning because of the fear that their muscle would then turn to fat.
2. If a strict rule is broken (or even disrupted) it may lead to negative mood states such as irritability, anxiety or guilt. Maintaining the exercise behaviour and continuing to follow the rule therefore means that these feelings are avoided.

Note: Scales 3 and 4 on the exercise profile (page 8) relate to behavioural rigidity: the higher the scores, the more rigid and compulsive the exercise behaviour is.

EATING DISORDERS, RIGIDITY AND COMPULSIVE EXERCISE (10 mins)

PERFECTIONISM

Share

Many people with eating disorders also experience some level of perfectionism. This refers to having a strong need for things to be done well or correctly. Perfectionism can lead to feeling bad if standards are not met, and the strength of the perfectionism varies from person to person. Some people may experience a small level of anxiety if something is not quite done right, whereas others may experience significant levels.

People who are perfectionistic often over-compensate by engaging in a compulsive behaviour to avoid feeling bad. If we relate this to exercise, following a strict rule means that you do it to a certain standard to avoid feeling bad. This means that the behaviour continues and becomes difficult to change or stop.

DICHOTOMOUS THINKING

Share

Many people with eating disorders can also be very rigid in their thinking, this is often referred to as 'black and white' or 'all or nothing' thinking. This means that you have an extreme view on defining things. For example, if it is not right then it must be wrong and ignoring everything in between.

Discuss

Can the group think of any other examples of 'all or nothing' thinking?

Prompt

If it is not good then it is bad, if it is not a success then it is a failure, if it is not 100% then it is a failure, if I am not underweight then I am overweight, if it's not beautiful then it's ugly.

Share

Strict rules are therefore a type of rigid thinking (all or nothing thinking). You either stick to the rule (which is right), or you don't (which is wrong). Unfortunately, this means that changing or breaking the exercise routine leads to feeling like you have 'failed' or let yourself down.

STRICT RULES (10 mins)

Share

Strict rules are usually expressed as 'I must…' or 'I should…' statements. They are often based on a certain standard or expectation (relate back to the perfectionism) and tend to be very rigid or inflexible (relate back to the dichotomous thinking).

Task

Ask the young people to note down any of their own exercise rules on page 30 in their handbooks. Ensure that they are phrased as 'I must…' or 'I should…' statements.

Share

Not all rules are unhealthy, even strict rules can be healthy. For example, you could have the rule "I must never exercise when I am injured", even if followed rigidly this rule would not lead to negative consequences or compulsive exercise.

Task

Handout laminated rules to each young person. Ask the young people to read out and divide the rules into columns of healthy and unhealthy rules.

Table 3: Answers for Healthy and Unhealthy Rule Statements

Healthy Rules	Unhealthy Rules
I must have rest days.	I must exercise no matter how tired or unwell I feel.
I should try to exercise with other people.	I must exercise every day.
I must regularly change my exercise routine.	I must burn off more than I have eaten.
I should always warm up and stretch my muscles.	I should always walk to school.
I must not push myself too hard.	I should always earn my food by exercising first.
I should only work within my capabilities.	I must do my exercises in the same order.
I must not burn more calories than I consume.	I should never miss an exercise session.
I should never exercise when I am injured.	I should always take the stairs instead of the lift.
	I must exercise at a certain time.
	I should always do more than I did last time.

SETTING NEW RULES (10 mins)

Task

Turn over the unhealthy rules and shuffle them up. Ask the young people to pick one out each. Go around the group and one by one decide whether this rule should be scrapped completely (cannot be followed without being unhealthy / leading to compulsive behaviour) or whether it can be adapted to form a more flexible, healthy rule.

Task

Ask the young people to note down one of their rules on page 31 in their handbooks, in the cost/benefit analysis table. Introduce the idea of evaluating their rules in terms of the costs and benefits (advantages and disadvantages) of breaking the rule (or scrapping it). There is an example of a completed table on the slides. Ask the young people to evaluate their own rule based on the costs and the benefits of breaking the rule, writing a conclusion and / or a new healthier rule underneath.

Discuss

How would you feel if you broke the rule? What would you gain if you didn't have to follow the rule?

SUMMARY (5-10 mins)

Discuss

Any Questions?

- What are the key things that you will be taking away from today?

Share

- People with eating disorders are often perfectionistic and rigid in their thinking, which leads to the setting and strict following of rules. Breaking a rule can lead to negative emotions.
- Because of this, behavioural rigidity is the most powerful maintaining factor for compulsive exercise.
- Not all strict rules are unhealthy.
- Healthy rules are much more flexible and do not lead to negative consequences or compulsive exercise.

Give the young people a blank Monitoring Record and Activity Questionnaire.

Session Six: Healthy Exercise and Urge Management

Resources

Session 6 PowerPoint, NEAT Handbook (Appendix 1.8), pens, highlighters, laminated formulation headings (Appendix 2.7), blue tac, whiteboard, whiteboard pens, laminated healthy vs unhealthy statements (Appendix 2.13), laminated exercise profile graph (x2) (Appendix 2.14), NEAT Exercise Monitoring Records (Appendix 2.5) NEAT Activity Questionnaire (Appendix 2.6)

REVIEW MONITORING RECORDS AND ACTIVITY QUESTIONNAIRES (5 mins)

Discuss

Go around each person in the group and ask them to share and reflect upon what they recorded in their monitoring records and activity questionnaires from Session 5:

- How have they found filling them out?
- Did they manage to resist any exercise urges?
- What did they notice, (ie; what worked to resist an urge, were there any similar themes that led to an urge?)
- If not completed, what are the reasons for this? Discuss ways in which the young people could overcome these obstacles.

Review activity challenge set in session 3: Did they complete the challenge to not attend a physical activity this week?

RECAP FORMULATION AND INTRODUCTION TO SESSION SIX (5 mins)

Task

Using a board and the laminated NEAT Formulation headings, ask the young people to get up and recreate the maintenance formulation for exercise. They can use blue tac to stick these up and the whiteboard pens to draw the relevant arrows.

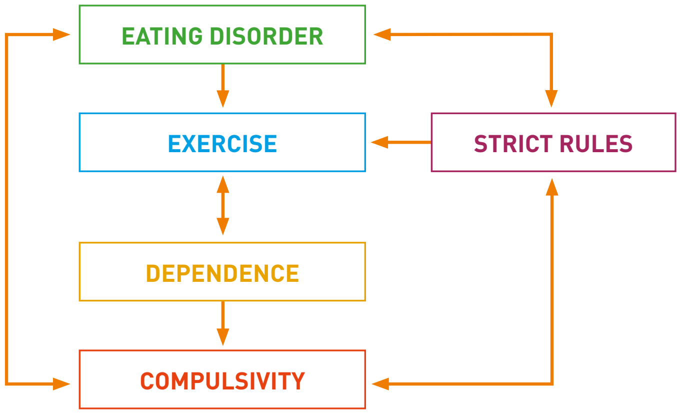

INTRODUCTION

Introduce session six and read out the aims.

- Session six will look at the difference between healthy and unhealthy exercise and introduce ways of managing urges to exercise.

By the end of session six you should know:

- The difference between healthy and unhealthy exercise.
- A range of techniques and ideas to manage exercise urges.

HEALTHY VS UNHEALTHY EXERCISE (10 mins)

Share

The concept of healthy and unhealthy exercise levels is not a rigid concept. Some guidelines suggest that one hour of exercise per day is a healthy level of exercise, however, this does not mean that anything over one hour becomes unhealthy. There are lots of factors to consider.

It is important to consider exercise on a continuum, with many factors to consider such as:

- Motivations for the exercise behaviour.
- The time of day that it takes place.
- Whether there is variety or a lack of variety (repetitive).
- The number of times that it is completed within a day or within a week.
- Rules that are set around exercise.
- The type of exercise engaged in.

Task

Handout the laminated healthy and unhealthy statements to each young person. Ask the young people to read out and split the statements into columns of healthy and unhealthy statements. Encourage discussion around their decisions and explore whether they can relate to any statements.

Table 4: Answers for Healthy and Unhealthy Statements.

Healthy statements	Unhealthy statements
I exercise because I enjoy it.	I use exercise as a means of earning certain privileges such as food.
I feel better about myself after I exercise.	I exercise primarily to change my weight or shape.
I use exercise to help me relax and destress.	I need to be slim in order to be fit.
I exercise in order to improve my health.	I am preoccupied with exercise; I think about it a lot.
I prefer exercise to be a social activity.	I exercise harder if I have done something wrong or been bad.
I follow a varied and flexible exercise routine.	I make up for any exercise sessions I miss by doing more next time.
I exercise to maintain a healthy weight.	I exercise to compensate for what I've eaten.
	I follow strict exercise routines.
	I make myself exercise even when I'm tired.
	I exercise in order to avoid feeling bad (e.g., anxious or irritable).
	I feel guilty about not exercising.
	I need to exercise in order to cope with how I feel.
	The fitter I am, the healthier I am.
	I prefer to exercise alone.
	I have exercise targets / goals that I must reach for it to be worth it.
	I feel that I have to exercise.
	I feel bad if I am unable to exercise (e.g., anxious, irritable, depressed or angry).
	I follow exercise rules – such as 'no pain, no gain' or 'more is better'.
	I exercise but I don't enjoy it.
	I continue to exercise even when I have an injury.
	I need to exercise in order to cope with life.
	I find exercise a chore and have to make myself do it.
	I continue to exercise when I am ill or not feeling well.

Discuss

So what makes exercise unhealthy?

Task

Ask the young people to note down their discussions on page 33 in their handbooks.

Prompts

Motivation: To influence weight and shape or to manage their mood (refer to the formulation).

Time of day: Before food to earn the calories or after food to burn off the calories (also known as debting), setting alarms to wake up early to fit in a routine, setting alarms to wake up in the middle of the night.

Number of times a day: Multiple times a day, engaging in multiple types of exercise/routines in spare time which allows little time for anything else, completing routines/exercise classes back-to-back.

Number of times a week: Not having a day off/7 days a week.

Variety: A lack of variety/engaging in repetitive routines.

Type of exercise: High intensity, lifting heavy weights, not working within capabilities, excessive incidental exercise, secretive exercise e.g. sit ups, squats, running on the spot.

Rules: Having very strict, inflexible rules.

Other points to consider: Not resting when ill or injured, exercising outdoors despite poor weather conditions, not fuelling the exercise/burning off more than you are consuming.

IDENTIFYING HEALTHY AND UNHEALTHY EXERCISE (10 mins)

Task

Split the group into two and ask the young people to read through the character stories starting on page 34 in their handbook and identify whether each character engages in healthy or unhealthy exercise. Provide the young people with highlighters so that they can highlight the reasons for their answer.

Ask the young people to also consider what scale(s) the characters may score high on the exercise profile. They can use the laminated exercise profile graph and white board pens to complete this visually, or just note down in their handbooks the most appropriate scales.

Table 5: Answers for Character Stories.

Walking Wendy: **Unhealthy**	• Rigidity: inflexible approach to exercise<br>• Fixed routine: repetitive and no variation<br>• Strict exercise rules<br>• Withdrawal symptoms: irritable, anger, anxiety<br>• Avoidant coping strategy: negative reinforcement<br>• No pleasure or enjoyment
Circuits Sam: **Unhealthy**	• Possible dependence on exercise: mood improvement and mood regulation<br>• Rigidity: interferes with social life and studies<br>• Fixed routine: repetitive, little variation<br>• Withdrawal symptoms: irritable, anger, depression<br>• Very obsessive attitudes: false beliefs about weight, shape, health, and fitness
Sporting Sue: **Healthy**	• Exercise is positively reinforcing: enjoyable, improves mood and no dependence<br>• Exercise is not rigid: happy to miss the odd session<br>• Routine is varied<br>• No obsessive attitudes despite weight loss being original motivating factor<br>• No emotional withdrawal symptoms
Random Rachel: **Unhealthy**	• Doesn't enjoy exercise at all, it's a chore<br>• Compulsivity: makes herself exercise even though doesn't want to<br>• Feels compelled to exercise due to guilt and worries<br>• Very obsessive attitudes with regards to weight and shape
Marathon Michelle: **Healthy**	• Exercise is positively reinforcing: enjoyable, improves mood, no indication of dependence<br>• Exercise is not rigid: misses the odd session and has rest days<br>• Exercise goals that are realistic and are not 'rules'.<br>• No obsessional attitudes or emotional withdrawal symptoms
Active Annie: **Unhealthy**	• Doesn't enjoy exercise at all – is a chore<br>• Makes herself exercise even though doesn't want to<br>• Feels compelled to exercise due to guilt and worries<br>• Obsessive attitudes about weight and shape

EXERCISE URGE MANAGEMENT (15 mins)

Share

The aim is to control your exercise and not be controlled by it. It is therefore important to learn ways of managing exercise urges.

MONITORING RECORDS

Share

The monitoring records that have been completed throughout the programme are an important tool to manage urges. They make you aware of urges and remind you that you have a choice about engaging in the behaviour. They also allow time to 'take stock' and challenge your thoughts.

For example: if someone's thought is that their weight is going to shoot up if they don't exercise, it allows time to consider previous weight data and whether this belief fits with the facts.

The monitoring records also allow you to identify times of day/situations/places that urges occur more frequently or intensely, which can encourage them to look for support during these times.

URGE SURFING

Share

Urge surfing refers to the use of distraction and relaxation to 'ride out' an urge. (Refer to graph on slides) The graph shows that over time the urge increases and reaches a peak. Many people believe that if you don't give in to the urge, then the intensity will remain high or increase further. In fact, if you can resist an urge to exercise you will find that it will become less strong without you needing to do anything other than wait. This is true for all urges and not unique to exercise. It is therefore important to think of ways to distract yourself, to support this process.

Task

Ask the young people to work as a group to identify distraction techniques that will support them in 'riding out' their urge to exercise and note these down on page 40 in their handbooks.

Share

The distraction needs to be:

- **Active:** it should involve you being actively engaged in something such as a task, rather than something passive like 'just watching whatever is on TV'.
- **Enjoyable:** it should not feel like a chore to do. The more you enjoy it, the more likely you are to engage in it exclusively and resist the urge to exercise.
- **Realistic:** something that you are likely to do or be able to do. It may be useful to think of distraction techniques for different situations / times of the day. For example, the distractions that you can use in the day may be very different to those you could use when you have an urge in the middle of the night.

For these reasons it is important that your distraction techniques are personal to you. Planning is also important when using distractions.

Task

Another way to ride out an urge is to use relaxation techniques. Relaxation enables you to focus on something unrelated to the exercise behaviour. Ask the young people to work together as a group to identify relaxation techniques that they could use. They can note them down on page 40 in their handbooks alongside their distractions.

SUMMARY (5-10 mins)

Discuss

Any Questions?

- Ask the young people to use and explore ways to manage their exercise urges further to reflect on in the final session.
- What are the key things that you will be taking away from today?

Share

What are the key things that you will be taking away from today?

- Defining healthy and unhealthy exercise is not clear cut. There are many factors to consider such as motivations to exercise, compulsivity, time of day etc.
- Having an understanding of the compulsive exercise formulation will help to determine whether exercise is healthy or not.
- The monitoring records completed throughout the programme are a useful tool for managing urges to exercise, by increasing awareness of them and allowing space for choice.
- Using distraction and relaxation allows you to 'ride out' the urge to exercise, known as the urge surfing technique.

Give the young people blank Monitoring Records and Activity Questionnaires.

Session Seven: Review and Reflection

Resources

Session 7 PowerPoint, pens, calculators, EDEQ (Appendix 2.1) and CET Questionnaires (Appendix 2.3).

INTRODUCTION

Introduce session seven and read out the aims.

- Session seven will look at the reflecting on the group programme and encouraging you to think further about your relationship with exercise.
- To reflect and review on NEAT.
- To explore whether the YP have utilised the group tools and found them helpful.
- To reflect on what the young people found helpful or unhelpful.

REVIEW USE OF MONITORING RECORDS AND URGE MANAGEMENT (10 mins)

TASK: Briefly reflect on monitoring records and whether anyone would like to share anything. The group do not need to share each urge, just any reflections they feel they've noticed over the week.

Discuss

What has been your experience so far using different tools to manage urges?

- Have the young people been utilising methods in their own time?
- How have they found the use of these?
- What works for them and what methods don't?
- Do they use the monitoring records in real time, and do they find this more helpful?

REVIEW OF SESSIONS (15 mins)

Task

Using the table below reflect on what we covered in each session and encourage the young people to share what they have learnt in each session. As you go through the sessions ask the young people to complete the formulation template in their handbook and encourage them to think about their individual experiences with each function of the formulation (page 43).

Table 6: Answers for Session Overview.

Exercise Profile Session:	• Discussed history of exercise and explored individual relationships with compulsive exercise.
Session 1 Introduction to NEAT:	• The maintenance formulation of compulsive exercise.<br>• The different definitions and the importance of including incidental exercise and physical activity when we think of exercise.
Session 2 Eating Disorder and Exercise:	• The different reasons why people exercise, in particular those with eating disorders.<br>• Explored the link between eating disorders and exercise and initiating and maintaining factors.<br>• Looked at activity anorexia and the biological and evolutionary theory to explain the link between the urge of being active at a low weight.
Session 3 Dependence:	• Explored how exercise dependence develops as a result of managing mood states.<br>• Positive and negative reinforcement – to feel positive emotions or get rid of feeling negative emotions.<br>• Similarities of exercise being addictive and the withdrawal symptoms that arise, which further increases dependency.
Session 4 Compulsivity:	• Beliefs and assumptions which lead to and maintain compulsive exercise.<br>• Often the compulsivity is driven by fears of what might happen if exercise stopped.<br>• Challenged these assumptions and whether there is evidence behind them or not.
Session 5 Strict Rules:	• The relationship between eating disorder, behavioural rigidity and compulsive exercise.<br>• Perfectionism and black and white thinking is present in eating disorders, therefore young people do not deviate from the strict rules they set themselves which maintains compulsivity.<br>• Deviating from strict rules also leads to negative emotions which further maintains exercise.<br>• Explored the differences between healthy and unhealthy rules.
Session 6 Healthy Exercise and Urge Management:	• Explored the differences between unhealthy and healthy exercise and ways to identify this.<br>• Discussed ways of managing exercise urges.

COMPLETE EXERCISE PROFILE (15 mins)

Share

The exercise profile helps you to formulate the reasons for exercise and the functions that exercise serves for you. We will re-complete this to explore whether there are any changes with your relationship with exercise or not.

Task

1. Ask the young person to work through each scale of the exercise profile in their handbook, ask them to answer based on the previous 2 weeks.
2. Once they have done this, ask them to calculate their scores and shade in their graph (page 51) to provide a visual representation of their exercise profile.
3. Ask the young people to look and compare their initial exercise profile (page 8) to this one and whether there are any changes or further things they could work on. They can look at the explanations in the book (page 9-11) to aid interpretation.
4. Ask the young people whether they want to share any of their observations. Explore with the young person how their exercise relates with their profile and any changes that have occurred.
5. Reassure the young people that there may still be things to work on and ways that they can do this.

RISK MANAGEMENT (10 mins)

Share

A risk management plan explores some of your triggers for compulsive exercise and ways to manage these triggers and urges after we've completed the group.

Task

Ask the young people to complete the risk management plan in their handbooks (page 53).

ENDING (5 mins)

Task

- Provide the young people with EDEQ and CET questionnaires to complete.

Discuss

- Any questions or reflections you would like to share with the group?
- Provide the young people with a pack of Monitoring Records and Activity Questionnaires templates if they wish to continue using after the sessions.

Note:

The young people can take their handbooks away with them.

Young Person's Handbook Information

The young person's handbook is used from session 0 to complete the exercise profile. At the beginning of each session, the handbook should be provided for the young people. The handbook includes different written activities that are completed within the sessions. Following the session material covered in the handbooks is a handout for the session. Practitioners should be aware that the handouts include material that is covered within the session. Therefore, practitioners should be mindful around young people referring to the handouts only once the session is complete. After each session, the handbook should be returned to the practitioner. Following completion of session 7, the young people can take the handbook away with them.

The young person's handbook has been separated for ease and reference into appendices. The practitioners should print the handbooks out as a collation for the young people. It is recommended that the practitioner also makes a copy of the handbook for their own reference to aid facilitation during the group.

My Exercise Profile

This activity is designed to help you understand the type of exerciser you are. The questions will help you identify not only your reasons for exercising but also the function(s) exercise serves for you.

Instructions: Read each question carefully and answer it as honestly as you can. Once you have answered the questions, follow the scoring instructions to calculate your mean (average) score for the scale before progressing to the next one.

Scale 1

If you are feeling low or depressed before you exercise, do you feel any happier (i.e., less depressed or low) after you exercise?	No, never<br>**0**	Yes, a bit happier<br>**1**	Yes, much, much happier<br>**2**
If you are feeling stressed or tense before you exercise, do you feel any less stressed or tense (i.e. more relaxed) after you exercise?	No, never<br>**0**	Yes, a bit less stressed, more relaxed<br>**1**	Yes, totally de-stressed, very relaxed<br>**2**
If you are feeling anxious before you exercise, do you feel any less anxious after you exercise?	No, never<br>**0**	Yes, a bit less anxious<br>**1**	Yes, totally anxiety free<br>**2**
If you have a lot on your mind before you exercise, do you find that the exercise helps you forget your worries/concerns?	No, never<br>**0**	Yes, a little<br>**1**	Yes, totally<br>**2**
Generally, how much of an effect would you say that exercise has on improving your mood (i.e. the way you feel)?	No, effect at all<br>**0**	A reasonable effect<br>**1**	A very large effect<br>**2**

Scoring: Add up (total) each column and add together, then divide the total by 5

**Column Totals:**			
**Grand Total (column totals added together):**			
**Grand Total divided by 5 (Mean Score):**		Round to one decimal place	

Scale 2

If you are unable to exercise, does it make you feel anxious because you cannot exercise?	No, never<br>**0**	Yes, a bit anxious<br>**1**	Yes, very anxious<br>**2**
If you are unable to exercise, do you ever feel frustratedand/or angry because you cannot exercise?	No, never<br>**0**	Sometimes/ occasionally<br>**1**	Usually or always<br>**2**
If you are unable to exercise, does it make you feel upset, low, or depressed because you cannot exercise?	No, never<br>**0**	Yes, a little bit<br>**1**	Yes, very much so<br>**2**
If you are unable to exercise, do you ever feel agitated and/or irritable because you cannot exercise?	No, never<br>**0**	Yes, a little<br>**1**	Usually or always<br>**2**
Do you feel like you need to exercise in order to avoid feeling any or all of the above (i.e., anxious, frustrated, upset, or irritable)?	No, never<br>**0**	A reasonable effect<br>**1**	Yes, very much so<br>**2**

Scoring: Add up (total) each column and add together, then divide the total by 5

**Column Totals:**			
**Grand Total (column totals added together):**			
**Grand Total divided by 5 (Mean Score):**		Round to one decimal place	

Scale 3

Do you try and follow definite rules regarding your exercise for example, a specific number of exercises, or a specific order in which the exercises must be completed?	No, not at all<br>**0**	Sometimes/ occasionally<br>**1**	Usually or always<br>**2**
Do you follow a set routine for your exercise sessions, such as always walk/run the same route, do the same exercises in the same order, spend the same amount of time and so on?	No, never<br>**0**	Sometimes/ occasionally<br>**1**	Usually or always<br>**2**
Would you describe your weekly pattern of exercise as repetitive?	No, not at all<br>**0**	Yes, a little<br>**1**	Yes, very<br>**2**
Do you set yourself exercise goals or targets that you feel you must reach?	No, not at all<br>**0**	Sometimes/ occasionally<br>**1**	Usually or always<br>**2**
Does it upset, annoy or irritate you when your exercise routine is interrupted or you are unable to follow a rule or meet your goals?	No, not at all<br>**0**	Yes, a little<br>**1**	Yes, very<br>**2**

Scoring: Add up (total) each column and add together, then divide the total by 5

**Column Totals:**			
**Grand Total (column totals added together):**			
**Grand Total divided by 5 (Mean Score):**		Round to one decimal place	

Scale 4

If you are unable to exercise, or you miss an exercise session, do you ever feel guilty?	No, never<br>**0**	Yes, a little guilty<br>**1**	Yes, very guilty<br>**2**
Does it worry you if you are unable to exercise, or when you miss an exercise session?	No, never<br>**0**	Yes, a little bit<br>**1**	Yes, a lot<br>**2**
If you miss an exercise session, do you try and make up for it next time you exercise (e.g. by doing more, or putting more effort in)?	No, never<br>**0**	Sometimes/ occasionally<br>**1**	Usually or always<br>**2**
Do you ever feel like you've let yourself down if you miss an exercise session?	No, never<br>**0**	Sometimes/ occasionally<br>**1**	Usually or always<br>**2**
Do you ever make yourself exercise even when you are tired, or do not feel like doing it?	No, never<br>**0**	Sometimes/ occasionally<br>**1**	Usually or always<br>**2**
If you are injured or ill, do you still continue to exercise?	No, never<br>**0**	Sometimes/ occasionally<br>**1**	Usually or always<br>**2**
Does your exercise ever interfere with your social life, work, or your study commitments?	No, never<br>**0**	Sometimes/ occasionally<br>**1**	Usually or always<br>**2**

Scoring: Add up (total) each column and add together, then divide the total by 7

**Column Totals:**			
**Grand Total (column totals added together):**			
**Grand Total divided by 7 (Mean Score):**		Round to one decimal place	

Scale 5

If you are unable to exercise, do you ever worry that you will gain weight, or get fat?	No, never<br>**0**	Sometimes/ occasionally<br>**1**	Usually or always<br>**2**
If you feel that you have eaten too much, do you ever do more exercise to burn it off/make up for it?	No, never<br>**0**	Sometimes/ occasionally<br>**1**	Usually or always<br>**2**
Do you exercise primarily to lose, rather than maintain your weight?	No, not at all<br>**0**	A little<br>**1**	Yes, absolutely<br>**2**
Do you exercise primarily to improve, rather than maintain your appearance and/or shape?	No, not at all<br>**0**	A little<br>**1**	Yes, absolutely<br>**2**

Scoring: Add up (total) each column and add together, then divide the total by 4

**Column Totals:**			
**Grand Total (column totals added together):**			
**Grand Total divided by 4 (Mean Score):**		Round to one decimal place	

Scale 6

	No, not at all / Not true at all	A little / A little true	Yes, absolutely / Totally true
Do you continue to exercise despite finding it a chore?	No, not at all<br>**0**	A little<br>**1**	Yes, absolutely<br>**2**
How true of you is it to say, "I do not really enjoy exercising"?	Not true at all<br>**0**	A little true<br>**1**	Totally true<br>**2**
Do you exercise primarily because you feel you have to, or need to, rather than because you enjoy it and want to?	No, not at all<br>**0**	A little<br>**1**	Yes, absolutely<br>**2**

Scoring: Add up (total) each column and add together, then divide the total by 3

**Column Totals:**			
**Grand Total (column totals added together):**			
**Grand Total divided by 3 (Mean Score):**		Round to one decimal place	

My Exercise Profile Graph

Using the mean score that you have calculated for each of the scales, draw a line on the graph and shade the area below the line. For example, if your mean score for scale 1 was 1.6, draw a line at 1.6 in column 1 of the table, and shade the area below the line. Do this for each of the scales.

**2**						
1.9						
1.8						
1.7						
1.6						
1.5						
1.4						
1.3						
1.2						
1.1						
**1**						
0.9						
0.8						
0.7						
0.6						
0.5						
0.4						
0.3						
0.2						
0.1						
**0**						
	Mood Improvement: Positive Reinforcement	Withdrawal Symptoms: Negative Reinforcement (Mood Avoidance)	Behavioural/Exercise Rigidity	Compulsive Exercise	Weight and Shape Exercise	Lack of Exercise Enjoyment
	**Scale 1**	**Scale 2**	**Scale 3**	**Scale 4**	**Scale 5**	**Scale 6**

Interpreting Your Exercise Profile

What does it all mean?

Below are descriptions for each of the scales, these will help you to understand your exercise profile and the things that maintain your exercise behaviour, as well as knowing the function(s) exercise serves for you.

Scale 1: Mood Improvement (Positive Reinforcement)

This scale measures how much of a positive effect exercise has on your mood.

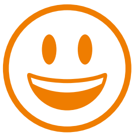

A high score on this scale shows that exercising leads to an improvement in your mood and you therefore find exercise very rewarding. This could put you at risk of becoming dependent on exercise to control your mood, and you may find it hard to cut down or stop your exercise behaviour.

A mid-range score shows that exercising leads to an improvement in your mood, but are unlikely to become dependent on exercise to control your mood.

A low score shows that exercise has little to no effect on your mood and it may be that exercise is not a very pleasurable activity for you.

Scale 2: Withdrawal Symptoms / Mood Avoidance (Negative Reinforcement)

This scale measures the effect on your mood if you are unable to exercise, and your dependence on exercise as a way of avoiding negative emotions.

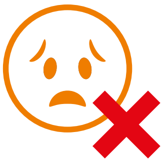

A high score on this scale shows that you use exercise as a way of avoiding a wide range of negative moods or emotions (e.g. depression, anxiety, irritability), and suggests a high level of dependence on exercise. This might mean that you find it very difficult to cut down or stop your exercise, because you worry about the negative impact on your mood (withdrawal symptoms).

A mid-range score could mean that you are either dependent on exercise as a way of avoiding negative mood states (e.g. anxiety or depression), or that you are 'at risk' of developing a dependence on exercise.

A low score shows that you do not use exercise to avoid feeling negative emotions and would be able to stop or cut down your exercise behaviour without suffering a negative mood state.

Scale 3: Rigidity

This scale measures how repetitive your exercise behaviour has become and how strict your rules for exercise are. Rigidity means unable to change.

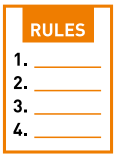

A high score on this scale shows that you may follow very specific 'exercise rules' and your exercise behaviour (routine) has become very rigid and repetitive. This makes it very difficult to cut down, stop, or even change your exercise behaviour.

A mid-range score shows that you like organisation and structure with your exercise but you do not need to follow specific rules. You would be able to cut down, stop, or change your exercise behaviour without too much difficulty, so long as it is carefully planned.

A low score shows that you can be flexible with your exercise, and you do not follow any sort of fixed routine or rules. You should be able to cut down, stop or change your exercise behaviour with little difficulty.

Scale 4: Compulsive Exercise.

This scale measures how compulsive you have become about your exercise. Compulsive means being unable to stop yourself.

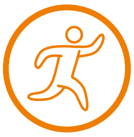

A high score on this scale shows that exercise has become so important to you that you feel you 'have to' exercise. You may hold false beliefs about the importance of exercise and you may worry what will happen to you if you stop or cut down. A high score therefore suggests that you would find it very difficult to cut down or stop your exercise behaviour.

This scale is one of the strongest indicators of an 'unhealthy' approach to exercise.

A mid-range score suggests a similar profile to a high-scorer only not as strong.

A low score suggests that exercise is viewed as important to you at an appropriate level, such as its importance to your general well-being.

Scale 5: Weight and Shape Exercise.

This scale measures how important exercise is to you, specifically in terms of its effect on your weight and shape.

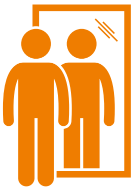

A high score on this scale shows that you exercise mainly for weight and shape reasons, and this could be maintaining your eating disorder. A high score may also suggest that you hold false beliefs about what 'healthy' exercise is, because exercise is only important for influencing shape and weight. Even in someone without an eating disorder, a high score on this scale would indicate an extremely unhealthy approach to exercise. You would find it very difficult to cut down or stop your exercise behaviour.

A mid-range score shows that you exercise largely for weight and shape reasons and you may hold some false beliefs about what 'healthy' exercise is. A low score shows that weight and shape is not your strongest reason for exercise.

Scale 6: Lack of Exercise Enjoyment.

This scale measures how un-enjoyable you find exercise.

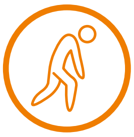

A high score on this scale shows that you do not enjoy exercise at all, and strongly suggests that you are exercising for the wrong reasons.

A mid-range score shows that although you do not always enjoy exercise, you do not hate it.

A low score on this scale shows that on the whole you find exercise an enjoyable activity.

A high or mid-range score on this scale as well as high scores on any of the other scales means that you need to re- learn what 'healthy' and enjoyable exercise is, so that you can continue to exercise in a way that is positive for your health and wellbeing.

Importance of My Exercise Profile

Your exercise profile is an important part of the NEAT treatment programme, a lot of what you will be taught will be specifically related to and best understood in terms of the scales you have just completed. Your exercise profile graph provides you with a visual image of your current relationship with exercise and will help you to identify what may need to addressed in order to return to 'healthy' and appropriate exercise.

Session One: Introduction to NEAT

What is NEAT?

- NEAT is a Cognitive Behavioural Therapy (CBT) group for compulsive exercise in Eating Disorders.
- The focus will be on the present and the future, and the factors that maintain the exercise behaviour.
- It will be a mix of slides, group discussions and tasks. There will be, at times, homework tasks set.
- NEAT is an active therapy group, which means that you will need to be able to take responsibility for behaviour change, with the information, guidance, support and encouragement from practitioners to help make these changes.

NEAT Aims

- To develop an understanding around what maintains compulsive exercise.
- To develop an understanding around the factors that might affect your attitude, beliefs and behaviours towards exercise.
- To understand the difference between 'healthy' and 'unhealthy' exercise.
- To introduce cognitive skills to challenge attitudes, beliefs and behaviours towards exercise.
- To introduce new coping strategies to help manage urges.

The overall aim is to help you gain CONTROL over your exercise and to have healthy attitudes, beliefs and behaviours towards exercise. It is not to stop you from exercising, but to equip you with knowledge and skills that will help them to regain control again.

Physical activity is:

....

....

Exercise is:

....

....

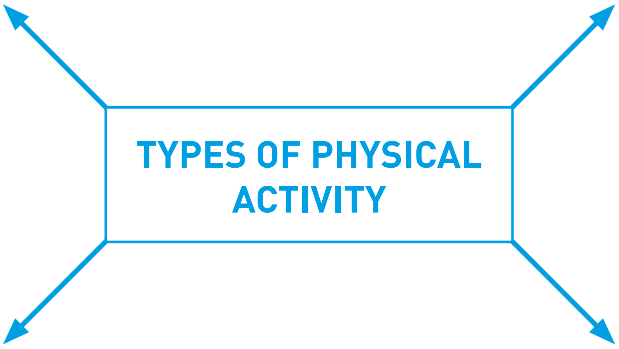

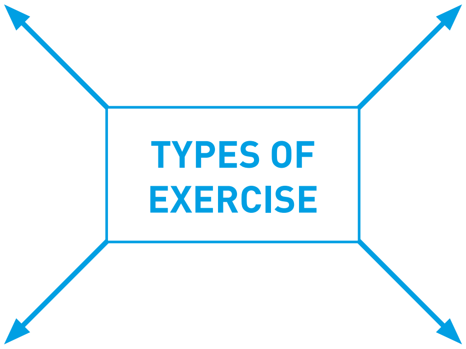

Incidental exercise is:

....

Examples:

....

....

Compulsive exercise is:

....

Introduction to NEAT

NEAT is a Cognitive Behavioural Therapy (CBT) for compulsive exercise.

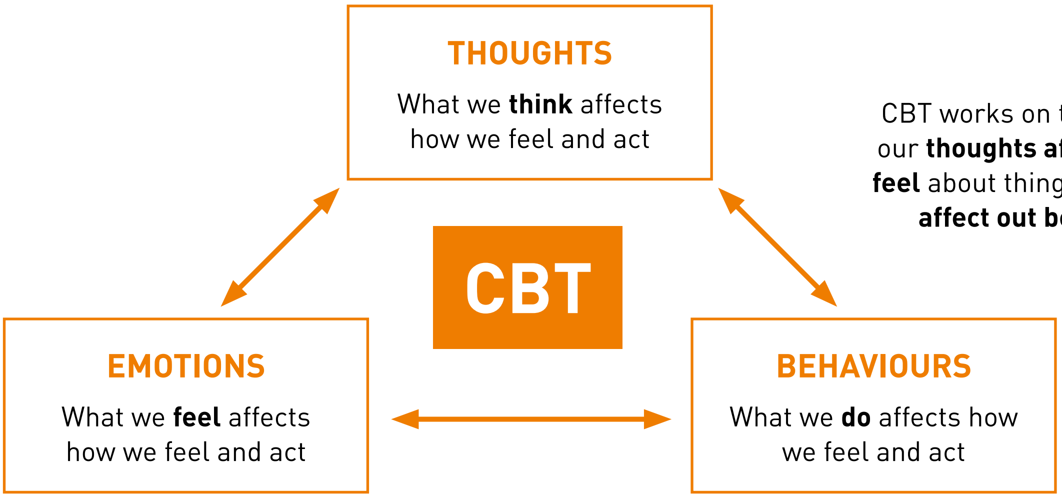

CBT works on the idea that our **thoughts affect how we feel** about things and in turn **affect out behaviour.**

The Exercise Maintenance Formulation

A formulation is a visual diagram that helps us to understand why a behaviour continues.
It breaks down the different factors that keep certain behaviours going.
Each session of NEAT will focus on a different section of the maintenance formulation for exercise.

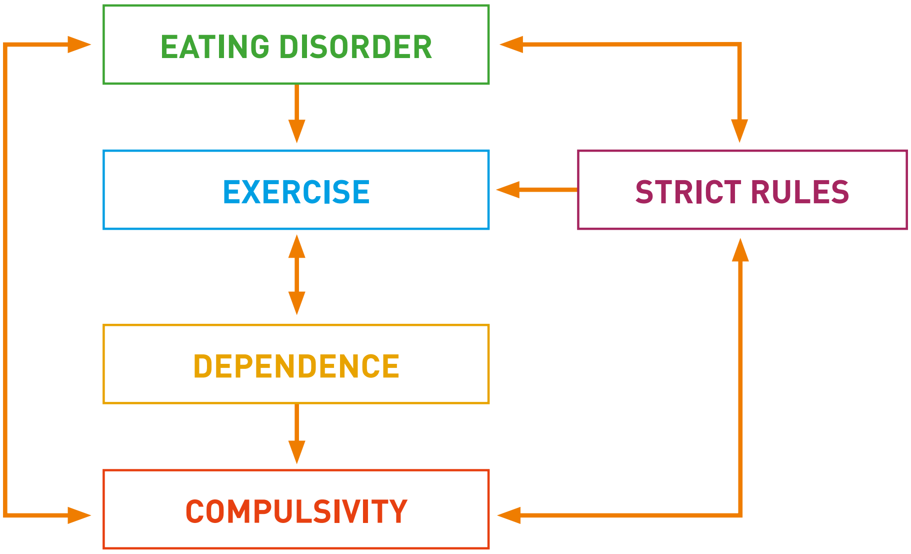

Session Two: Eating Disorders and Exercise

Initiating and Maintaining Factors

What are Initiating and Maintaining Factors?

Initiating factors = reasons for starting a behaviour.

Maintaining factors = reasons for not stopping the behaviour.

It may be that the reason you started exercising was to get fit and because you enjoyed it, but the reason you cannot or don't want to stop exercising is because you are afraid you will gain weight. Therefore, the reason you started is not the same as the reason you cannot or don't want to stop. Using the space below, write down the reasons you first started exercise.

Why I started exercising

It is important to understand that when you first start a behaviour, such as exercise, your initiating and maintaining factors are likely to be the same. For example, if you started exercising to get fit, then it is likely that the reason you kept exercising was to stay fit. However, over time it is possible for the factors maintaining your behaviour to change, sometimes without you being aware. In order to change your behaviour, it is important that you identify the things that keep it going.

How to Identify Maintaining Factors.

One way to identify the factors that maintain your exercise behaviour is to look at what you get out of exercise, e.g. its function. Your exercise profile should have helped you to identify some of the main functions exercise serves for you. For example, to avoid certain emotions or to control weight and shape (refer back to your profile on page 8).

Using your Exercise Profile as a guide, try and identify some of the main functions exercise serves for you and write them below.

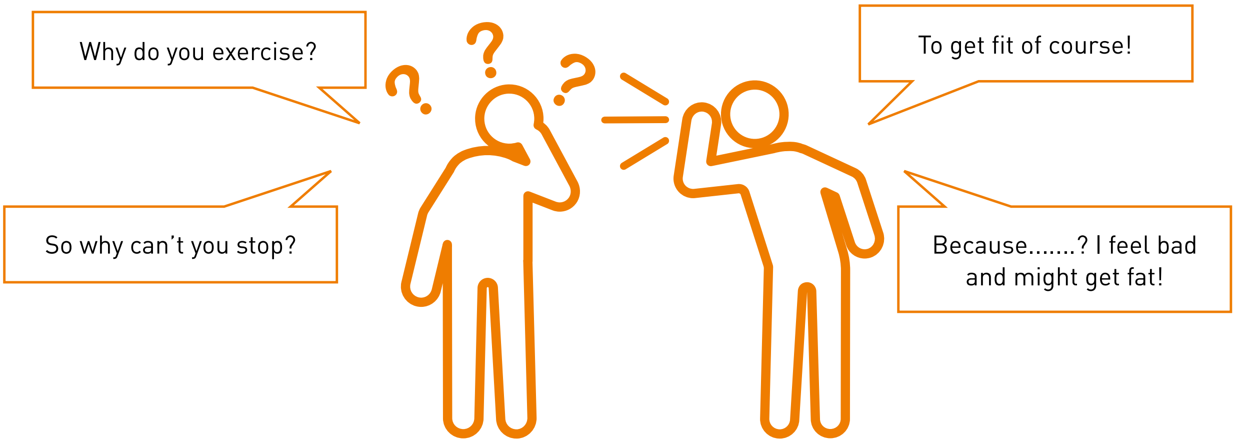

The function of my exercise

Fear of Negative Consequences

Another way to identify which factors are maintaining your exercise behaviour is to look at what you're afraid may happen if you stopped exercising. This fear makes you continue to exercise so that you can avoid the negative consequences.

You can get a good idea of whether a fear of negative consequences is a maintaining factor for you by looking at your score on scale 4 of your Exercise Profile. This scale measures compulsivity. Compulsive exercise can be a result of the fears you have if you stopped exercising.

Using the space below, write down any fears or concerns you may have about stopping or reducing your exercise. A tip here is that some of your fears may be related to the function exercise is serving for you (e.g. avoidance).

My fears

Eating Disorders and Exercise

Weight and Shape

1. Some people exercise purely as a way of controlling their weight and shape, and only started exercising after they developed weight and shape concerns as part of their eating disorder.
2. Some people were exercising healthily before the eating disorder took over their exercise as a way of controlling weight and shape.
3. Some people's exercise is unrelated to their eating disorder and they continue with what they were doing before the onset of the eating disorder.

The eating disorder can often use exercise to control weight and shape and this can happen without you being aware, especially for people who were exercising before the onset of the eating disorder.

The following short questionnaire will help you to assess whether you have exercise attitudes, beliefs and behaviours that are influenced by the eating disorder.

		YES	NO
1.	Do you ever adjust the amount of exercise you do according to what you have eaten?	☐	☐
2.	Do you worry that you will gain weight if you do not exercise?	☐	☐
3.	Do you ever work out how much you can eat based on how many calories you have burned or can burn?	☐	☐
4.	Do you ever feel the need or desire to exercise after you have eaten?	☐	☐
5.	Do you ever exercise after a binge?	☐	☐
6.	Do you ever use exercise as a means to earn the right to eat?	☐	☐
7.	Do you ever use exercise to manage any guilt you feel after eating?	☐	☐
8.	Do you ever think about how many calories you are burning when you are exercising?	☐	☐
9.	Do you ever weigh yourself before and after exercising to see if you have lost weight.	☐	☐
10.	Do you feel thinner after you exercise?	☐	☐
11.	Do you exercise primarily to lose weight?	☐	☐
12.	Do you ever do more exercise as a punishment for eating certain foods or eating too much?	☐	☐
13.	Do you exercise primarily to improve your appearance?	☐	☐
14.	Did your interest in exercise begin with a desire to lose weight?	☐	☐
15.	Do you ever measure your “self-worth” in terms of how much or how well you exercise?	☐	☐
16.	Have you ever exercised in secret?	☐	☐

Your score does not show how severe your eating disorder is, it simply looks at the relationship between your exercise beliefs and behaviours and your exercise. The more YES’s you recorded, the stronger this relationship is. This may suggest that you exercise because of weight and shape concerns.

Activity Anorexia

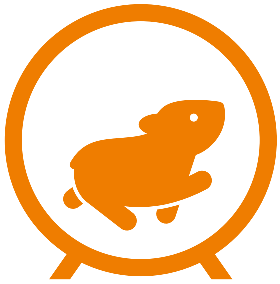

2 groups of rats were given an exercise wheel.

Group 1 were given enough food to stay a healthy body weight.

Group 2 were not given enough food and the rats lost weight.

The rats were then watched to see what they would do.

Did group 1's activity increase, decrease or stay the same?

....

Did group 2's activity increase, decrease or stay the same?

....

Why?

Biological Theory

The urge to be active is controlled by a brain chemical called ....

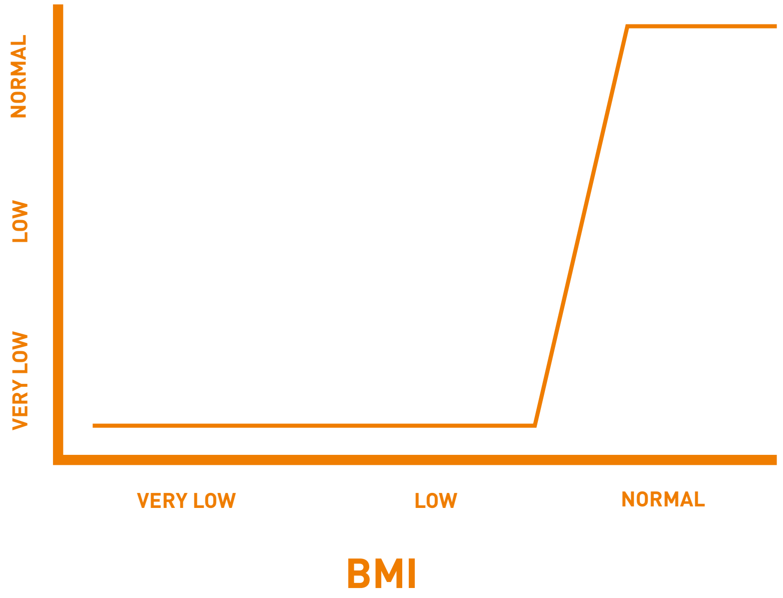

The graph shows that a low weight results in low levels of...

This means that the person will experience an increase in the urge to be active, and will not receive a signal to stop when the body has done enough. It is only when the body returns to a normal weight that the brain chemical levels will normalise.

Evolutionary Theory

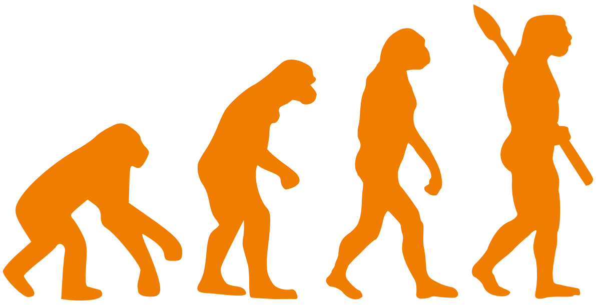

Humans have an urge to be active when they do not have enough food because...

....

....

....

....

Eating Disorders and Exercise

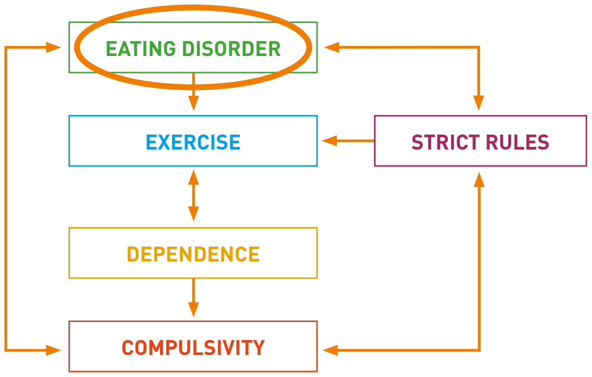

People exercise for many reasons:

- Because it's healthy
- To keep fit
- To socialise
- As a hobby
- To compete against others
- For fun
- For achievement

However, the reason that someone may start exercising (initiating factors) is often not the reason they cannot stop exercising (maintaining factors).

Exercise is a healthy, life improving activity that should be enjoyed.

When someone has an eating disorder, the reasons for starting to exercise may be different and the risk of the exercise becoming compulsive increases.

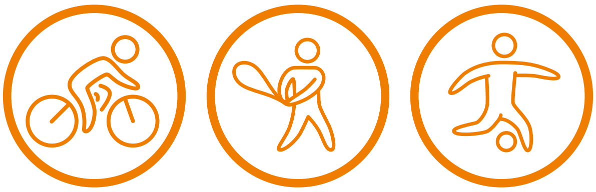

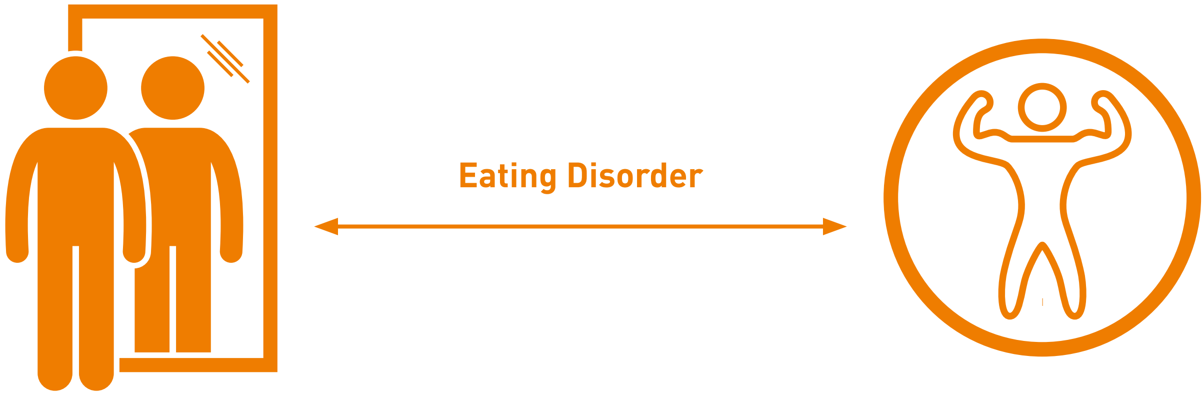

Weight and shape concern

Increased urge to be active

Session Three: Exercise Dependence

Exercise is a very powerful way of changing how you feel. Even a small amount of exercise, as little as 20 minutes of mild activity, has been shown to improve mood or make people feel less anxious or low.

How much of an effect does exercise have on improving your mood?

None at all |————————————————| **Very large**

The benefits from exercise happen in the first 20 minutes of mild exercise. Intense exercise does NOT have the same positive effect. In fact, it often results in a negative mood.

'Addiction'

The powerful effect on mood can, over time, lead to a psychological dependence on exercise (a strong desire to continue). This experience is sometimes referred to as an 'addiction'.

It is not possible to become physically addicted to exercise, however, the psychological dependence can feel so strong that it feels like a real addiction.

To what extent do you need to exercise in order to feel good?

None at all |————————————————| **Always**

Would you consider yourself 'addicted' to exercise

None at all |————————————————| **Totally**

Emotional Withdrawal Symptoms

When someone cannot exercise, they may experience negative mood changes such as anxiety, anger, low mood, irritability and frustration. This makes it very difficult to stop or cut down exercise, as continuing to exercise means that you can avoid these negative emotions.

Exercising to avoid feeling negative emotions is more powerful in keeping your exercise behaviour going than feeling better after exercise.

How easy do you find it to stop exercising altogether?

Easy |————————————| **Impossible**

To what extent do you continue to exercise in order to avoid feeling anxious, irritable, depressed, frustrated, angry, or upset?

Not at all |————————————| **Always**

Exercise Restriction

During your treatment, it is advised activity is reduced to a minimal level. Why do we do this?

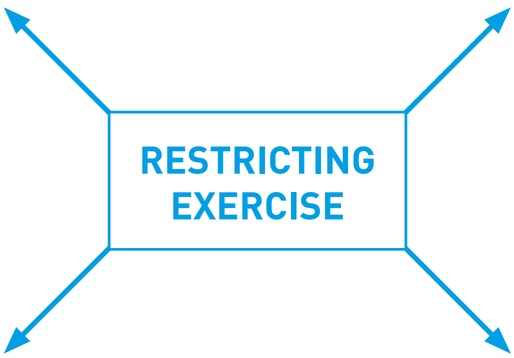

Exercise Dependence

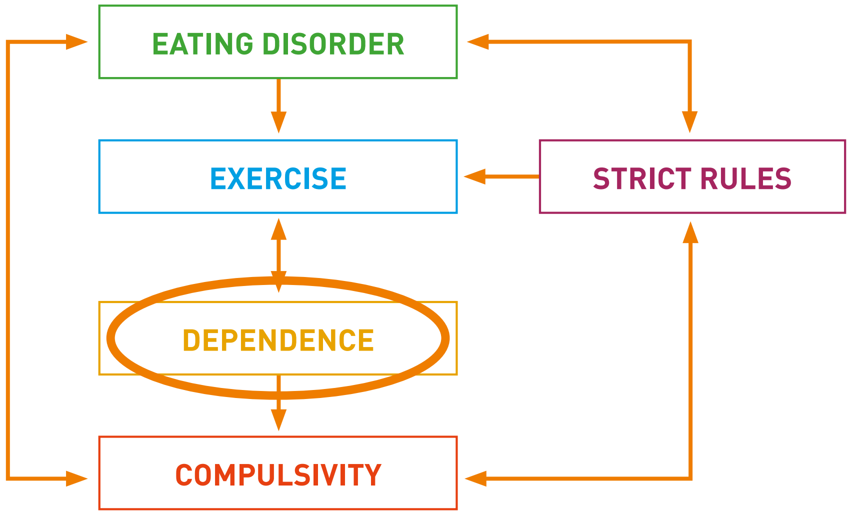

Dependence on exercise is related to 'mood regulation' (the ability to manage our mood).

If a person has difficulty managing their mood, they may use exercise to do this, because it has a powerful effect on mood.

Positive Reinforcement
(something positive being added)
Exercising can improve your mood and make you feel more energised / happy.

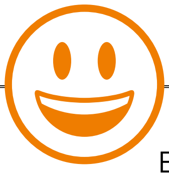

Negative Reinforcement
(something negative being taken away)
If you are feeling low / anxious / stressed, then exercising may take these feelings away.

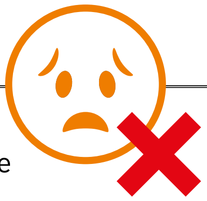

Both increase the chances of exercise behaviour being repeated!

When someone cannot exercise, they may feel anxious, irritable or low. This is known as emotional withdrawal symptoms. This makes it very difficult to stop exercising, because you continue to exercise to avoid these negative feelings.

Dependence on exercise can feel a lot like a real addiction. Although it is not possible to become physically addicted to exercise, it is important to note that the treatment of compulsive exercise is the same as an addiction – complete abstinence, learning new coping strategies and gaining control over the behaviour.

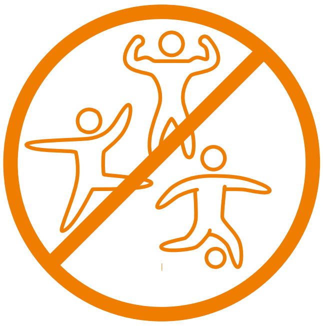

Session Four: Compulsivity

Myths and Facts

Compulsivity does not refer to how much or how often.

If a behaviour cannot be easily stopped or changed, then it is considered compulsive.

		MYTH False	FACT True
1.	All body fat is unhealthy	☐	☐
2.	Exercise can change/regulate your mood	☐	☐
3.	Muscle weighs approximately three times as much as fat	☐	☐
4.	Walking to the shops or train station is not exercise	☐	☐
5.	You need to be thin to be healthy	☐	☐
6.	The thinner you are the more obsessional and rigid you become	☐	☐
7.	Fat can be turned into muscle	☐	☐
8.	If you are fit, then you are healthy	☐	☐
9.	Exercise can be addictive	☐	☐
10.	You need to be thin in order to be fit	☐	☐
11.	The human body is naturally thin	☐	☐
12.	If a muscle is not used, then it turns to fat	☐	☐
13.	You cannot be fat and fit	☐	☐
14.	It takes less exercise to maintain fitness than it does to improve it	☐	☐

Fears about what will happen if we stop exercising can often be based on myths that we assume are true. These myths can therefore contribute to the maintenance of the exercise behaviour, because they reinforce the irrational belief.

The Body Builder Analogy:

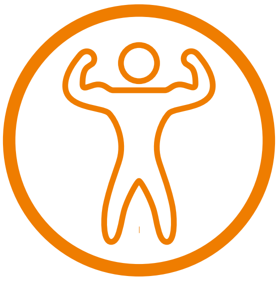

Imagine a bodybuilder, it is usually a great big muscular guy or girl with huge arms, legs and chest muscles that are much bigger than normal.

When a bodybuilder has a show or competition coming up, they need to prepare their bodies so that all their muscles are really well defined and hard, and the judges can see all the muscular definition. In order to get that sort of definition, a bodybuilder will dehydrate their bodies to dangerous levels and change their diet so that their bodyfat percentage drops to around **5%**.

Now, imagine that the bodybuilder stopped training - stopped lifting any weights, but changed their diet so that their body fat percentage stayed the same at **5%**.

After a while, what would they look like – all hard, toned and defined, or soft and undefined?

The answer is that they would look soft and undefined, **BUT** their bodyfat percentage has stayed the same at **5%**. Their muscle cannot have turned to fat – so what has happened?

> Unused muscle relaxes, but it does not turn to fat (it is still muscle just not hard). Muscle and fat are two completely different sorts of tissue, meaning that one cannot turn into the other.

Bodyfat is so important for the body to work properly, and when someone becomes really underweight, the body starts to break down muscle tissue instead of fat because keeping a reasonable level of fat is more important for survival than keeping muscle.

What's True and What's Not?

Your beliefs

What do you believe to be true about exercise? Try to think of things that you have heard but are not too sure of, they could be about the benefits of exercising, the consequences of not or the significance of incidental exercise.

....

....

....

....

....

Your Assumptions

What assumptions do you have about exercise? They may be personal assumptions based on your own behaviour (e.g. if I... then...) or more general assumptions based on what you believe to be true for people on the whole (e.g. if people... then people will...).

If .... then ....

If .... then ....

If .... then ....

If .... then ....

If .... then ....

Is my belief a myth or a fact?

My belief about exercise is:

Evidence to support this belief	Evidence against this belief

If your belief is a fact, then there should be more information in the evidence to support column. But, if there is more evidence against your belief, it could be based on a myth / assumption.

**My conclusion** (i.e. my belief is not completely true because... / my belief is completely true because.../ this means that...)

Compulsivity

Compulsivity does not refer to how much or how often.

If a behaviour cannot be easily stopped or changed, then it is considered compulsive.

Fears about what will happen if we stop exercising can often be based on myths that we assume to be true.

Our exercise can therefore be maintained by a myth that has very little evidence to support it.

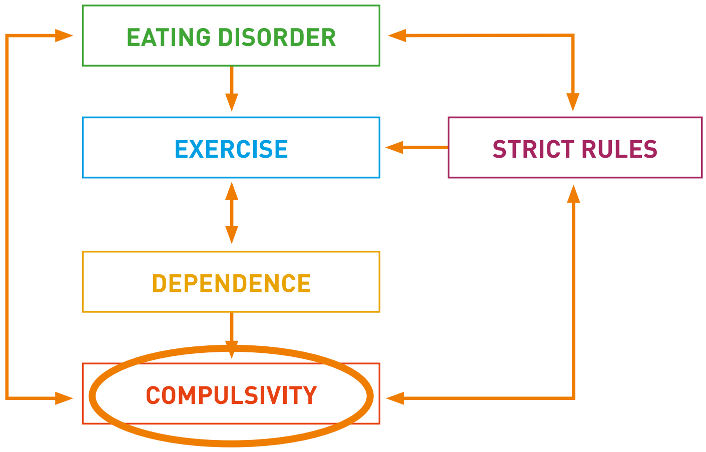

What is the difference between a myth and a fact?

- A myth is a popular belief that is false or unsupported by any evidence.
- Facts can be supported by evidence.

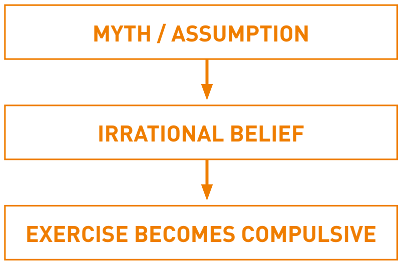

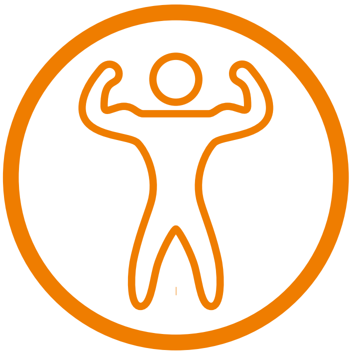

Lots of common myths have come from trying to make sense of a relationship observed e.g. "if I don't exercise then my muscle will turn to fat"

However, scientific evidence shows us that fat and muscles are completely different types of tissue, so one cannot become the other.

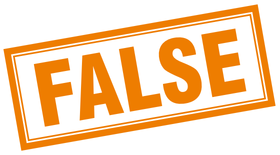

You can test your beliefs by looking at the evidence for and against to decide whether it is based on a myth or a fact. This should help to reduce some of your fears about stopping or changing your exercise behaviour.

Session Five: Strict Rules

Strict rules include following specific routines or orders of events. Rules are strict if they are inflexible and hard to stop, or cause negative emotions if the routine is disrupted or broken.

Strict rules (and being very rigid) is one of the most powerful maintaining factors for compulsive exercise, because it makes it difficult to stop or cut down the exercise behaviour.

Strict rules are usually expressed as 'I must...' or 'I should...' statements, and are usually very rigid and based on high expectations or standards.

Note down some or all of the exercise rules that you currently follow or have followed in the past.

My Rules

....

....

....

....

....

....

....

Not all strict rules are unhealthy, even if followed rigidly. For example, "I must never exercise when I am injured" would not lead to negative consequences or compulsive exercise. Healthy rules are more flexible and more like guidelines, which makes them easier to follow.

Cost / Benefit Analysis

My rule is:

Costs of breaking the rule	Benefits of breaking the rule

**My conclusion**<br>(i.e. there are more costs / benefits to breaking my rule, my rule could be adapted to be more of a guideline, my rule needs to be scrapped, I can continue to follow my rule etc.)

My new rule (if applicable) ....

Strict Rules

The Exercise Maintenance Formulation

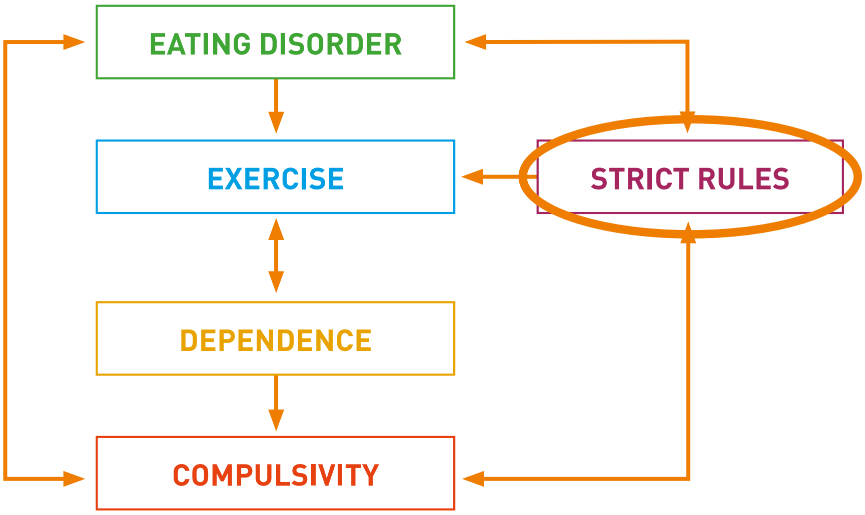

Strict rules include following specific routines or order of events.

You may feel negative emotions if this routine is stopped or disrupted, such as anxiety or anger.

Strict rules may be set because of strong beliefs about what will happen if you stop exercising.

They are usually expressed as 'I must…' or 'I should…' statements.

Eating Disorders and Strict Rules

Many young people with eating disorders experience some level of perfectionism – a strong need to do things well.

Strict rules are therefore often based on set standards or expectations, and must be followed perfectly.

Many people with eating disorders can also be very rigid in their thinking (inflexible). This means that you have an extreme view on defining things.

for example – 'if it is not black then it is white'

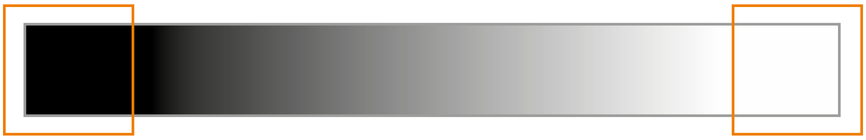

and ignoring all of the shades of grey in between!

Strict rules are therefore inflexible, they may be specific routines done in specific orders, and again these must be followed perfectly.

However, not all strict rules are unhealthy

Session Six: Healthy Exercise

The concept of healthy and unhealthy levels of exercise is not a rigid, black and white concept.

Some suggest that one hour of exercise per day is considered healthy, but this does not mean that anything over one hour becomes unhealthy. You have to consider many different factors to fill in the grey area in between!

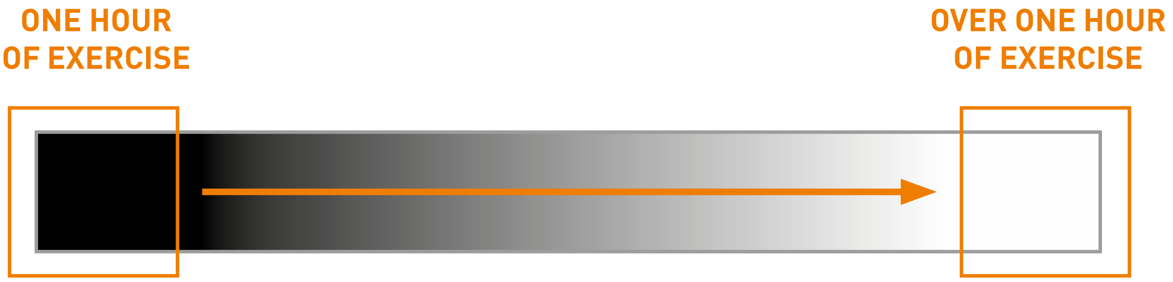

What makes exercise unhealthy?

Motivation: ....

Time of day: ....

Frequency in the day: ....

Frequency in the day: ....

Frequency in the day: ....

Variety: ....

Time of day of exercise: ....

Rules: ....

Identifying healthy and unhealthy exercise

Walking Wendy

Wendy is 22, works part-time at a shop and is a regular walker. Wendy always walks the same route at the same time every day, which only takes her about 45 minutes at a gentle pace. Wendy's walk isn't motivated by weight or shape concerns as she never changes her walk if her diet changes and never tries to make up for any missed walks. In fact, Wendy doesn't even start to sweat and she never sets herself any exercise targets. However, Wendy does follow strict exercise rules. She cannot change her route, or the time she goes for her walk, nor can she reduce the time or distance, or miss the walk for a day without feeling extremely anxious. Nor can Wendy tolerate being interrupted when she is walking as it makes her irritable and angry. Wendy doesn't feel any 'better' or 'happier' after her walk as she doesn't really enjoy it, she just feels less anxious.

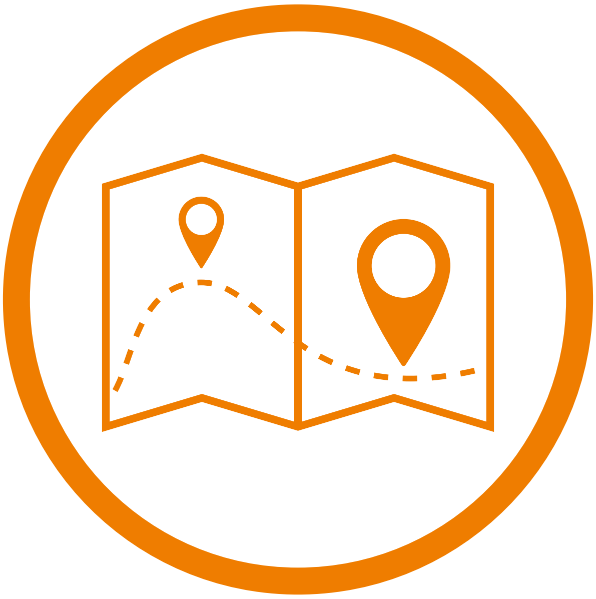

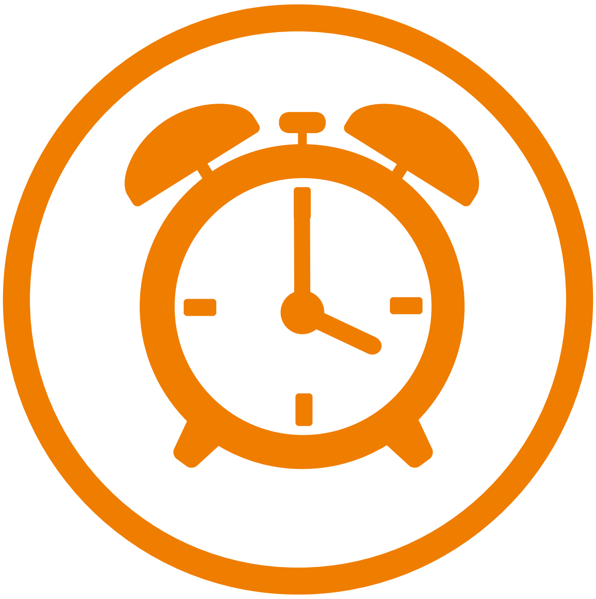

Is Wendy a healthy exerciser or unhealthy exerciser?

Healthy ☐ Unhealthy ☐

What scale(s) of the exercise profile may Wendy score high on?

....

....

Circuits Sam

Samantha is 19, a student and is a fitness fanatic. She attends the same circuit's class at a gym 5 times a week and goes for a run every day at the same time before she goes to university. She also does a yoga class at the weekend and will do extra gym classes if she has nothing else to do. Samantha always feels much happier after she has exercised and finds it really boosts her mood. Samantha believes that she needs to exercise every day in order to stay slim and that the fitter she is, the healthier she is. In order to make herself exercise as hard as she can, Samantha sets herself targets to reach at the gym, such as doing more repetitions or lifting more weight. She also sets running targets, such as running further in the same time or running the same distance but faster. If she cannot reach one of her targets, Samantha feels like she's let herself down and that her day has been ruined. Because Samantha believes that fitness and slimness are related she watches what she eats in order to not gain any weight and will often do more exercise to make up for eating a bad food or too much food. If Samantha cannot exercise for any reason she feels irritable and annoyed, and a bit low/depressed. She also worries that she will lose fitness and gain weight and will make up for any exercise she has missed by doing more next time. Samantha often feels she isn't doing enough, or could be doing more exercise, but it is difficult to fit any more in as it is already affecting her studies. If she has a lecture at the same time as her circuits class she will miss the lecture, and she often turns down invitations to parties as it will mean she misses an exercise session.

Is Sam a healthy exerciser or unhealthy exerciser?

Healthy ☐ Unhealthy ☐

What scale(s) of the exercise profile may Sam score high on?

....

....

Sporting Sue

Sue is 26, a full-time mum and is an active member of several sports teams. Sue never used to play any sport or do any other exercise, but she became worried that she was putting on weight and decided to do some exercise to help her lose some weight. Sue initially joined a gym but found it too boring, so she joined her local women's football team. Sue found she enjoyed the social side of team sport a lot and now plays football 3 times a week as well as playing tennis 2-3 times a week. Despite starting exercise to lose weight it is now the social aspect and the enjoyment Sue gets from it that keeps her going. Sue also likes walking and often goes for long walks in the countryside or along the coast with her friends and family. Sue always feels a lot better after she has done some exercise and finds that it helps her to relax and unwind from a stressful day. Sue often misses one of her training sessions due to other commitments, or sometimes just because she can't be bothered, but so long as she makes it most of the time she feels that it is ok.

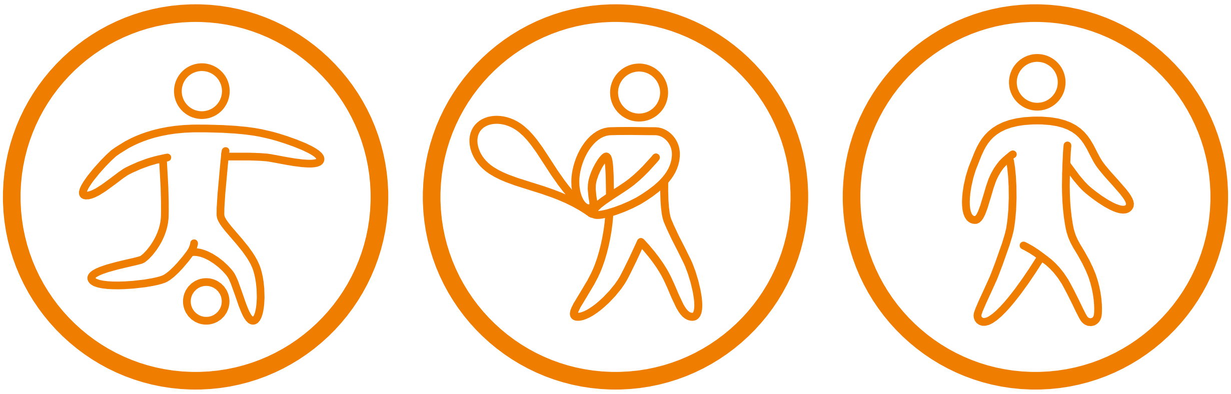

Is Sue a healthy exerciser or unhealthy exerciser?

Healthy ☐ Unhealthy ☐

What scale(s) of the exercise profile may Sue score high on?

....

....

Random Rachel

She always hated sport and is naturally not that active as a person. Rachel likes walking round the shops and doesn't mind walking to the train station or somewhere locally, but she doesn't enjoy gyms or exercise classes. Lots of Rachel's friends are regular exercisers, are members of the gym and are very concerned about their weight and shape. Rachel didn't used to be bothered about her weight or shape as she is naturally quite slim albeit curvy, but since her friends are now all slimmer than her Rachel worries that she is fat. As a result, Rachel often feels really guilty about the amount she has eaten and will do 1, 2, or even 3 hours of exercise to compensate. When Rachel feels the need to exercise she has to do it immediately even if she feels tired or has other commitments. If she cannot exercise, Rachel feels guilty that she has let herself down. Rachel doesn't follow any sort of exercise routine and will often not exercise for several days, but when she does exercise she tends to do a lot, usually running, sit-ups and press-ups. Rachel's friends keep trying to get her to come to the gym with them, but she doesn't want to as she feels she would be the fattest person in the gym because all her friends are so thin. Rachel often joins her friends on various diets, but she finds them impossible to keep to and ends up exercising to make up for breaking the diet.

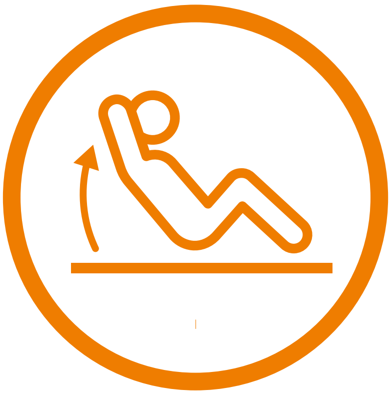

Is Rachel a healthy exerciser or unhealthy exerciser?

Healthy ☐ Unhealthy ☐

What scale(s) of the exercise profile may Rachel score high on?

....

....

Marathon Michelle

Michelle is 21, works in an office and is training for a marathon. Michelle has always enjoyed running since she started cross country running at school when she was 12. Although she used to compete in local and national competitions, Michelle does not compete any more. However, Michelle is very competitive by nature and highly perfectionistic, so she always strives to do the best she possibly can. She is following a strict training routine, which she got from a running magazine, that is specific for running a marathon and slowly builds up her weekly mileage. The training routine also incorporates weekly performance goals that should be aimed for. Although the training routine is strict, each week is different and always incorporates 2 or 3 rest days. Michelle follows the prescribed routine exactly and never tries to do any more. On average Michelle is doing about 7-8 hours of exercise a week. Michelle is also following a fairly strict diet. She weighs herself several times a week to make sure that she is maintaining her weight and she increases her calorific intake each week to make up for the extra exercise. Michelle is aware that her running keeps her slim and in good shape, and she also likes the way running makes her feel, but she knows that too much exercise can damage her health so several times a year she will stop all exercise for a week or two to give her body a rest. Although she misses running when she stops, Michelle doesn't worry or feel guilty and isn't concerned that she'll gain weight or lose shape.

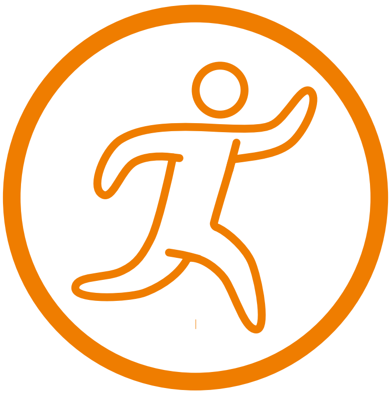

Is Michelle a healthy exerciser or unhealthy exerciser?

Healthy ☐ Unhealthy ☐

What scale(s) of the exercise profile may Michelle score high on?

....

....

Active Annie

Annie is 21, works as a groom at a stable yard and is a regular gym user. Despite having a physical job Annie goes to the gym 5 times a week for an hour after work, in order to stay slim and in shape. Annie finds the gym a real chore as she doesn't really enjoy it, but feels she has to do it if she wants to stay slim. She makes herself go even though she is often exhausted after a hard day at work. If Annie is unable to go to the gym for whatever reason she always feels really guilty about missing it, like she's let herself down. She also worries that she will become unattractive if she doesn't keep going to the gym as she believes that she needs to exercise in order to be attractive. Annie is always looking at the other people in the gym, comparing herself to them and thinking that they are slimmer and more attractive than her. She also notices that they seem to have more energy than her and she thinks this is because they are fitter than her. Annie thinks that the slimmer you are the more attractive you are and that if she were slimmer she would feel a lot better.

Is Annie a healthy exerciser or unhealthy exerciser?

Healthy ☐ Unhealthy ☐

What scale(s) of the exercise profile may Annie score high on?

....

....

Managing Exercise Urges

The aim is to control your exercise and not be controlled by it, it is therefore important to learn ways of managing your exercise urges.

Urge Surfing

If you can resist an urge to exercise, you will find that it becomes less strong without you needing to do anything other than wait. Urge surfing refers to the use of distraction and relaxation to 'ride out' an urge.

Distractions need to be:

1. Active – you should be actively engaged in the distraction.
2. Enjoyable – it should not feel like a chore.
3. Realistic – something that you are able to do (it may be useful to think of different techniques for different situations).

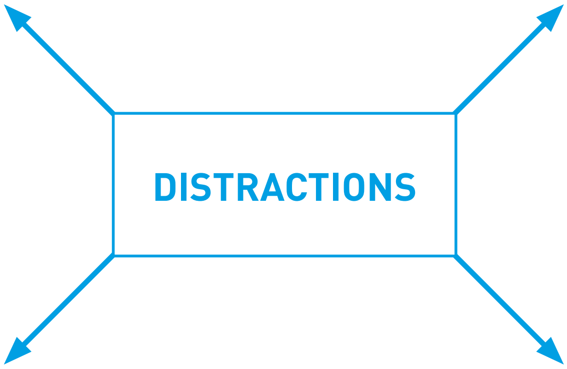

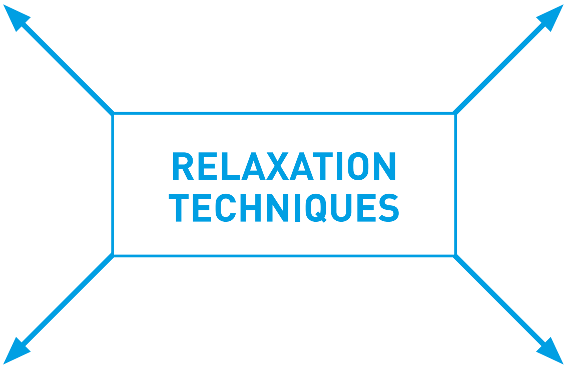

Healthy Exercise

Defining healthy and unhealthy exercise is not a rigid, black and white concept. Some suggest that 1 hour of exercise per day is healthy, but that doesn't mean that anything over 1 hour becomes unhealthy.

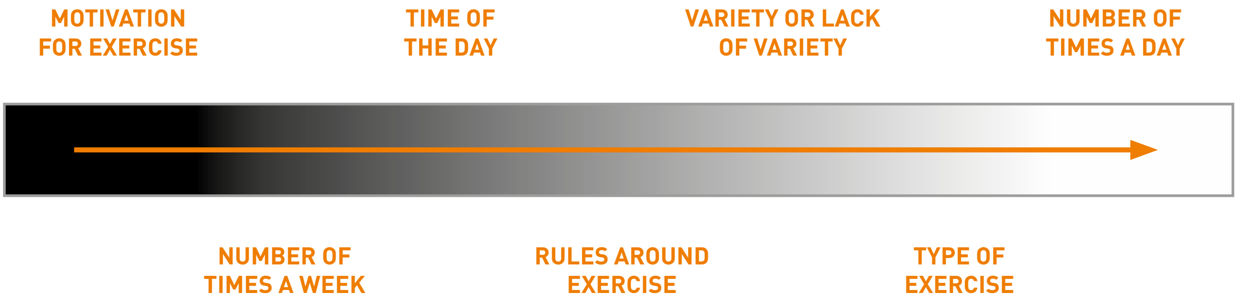

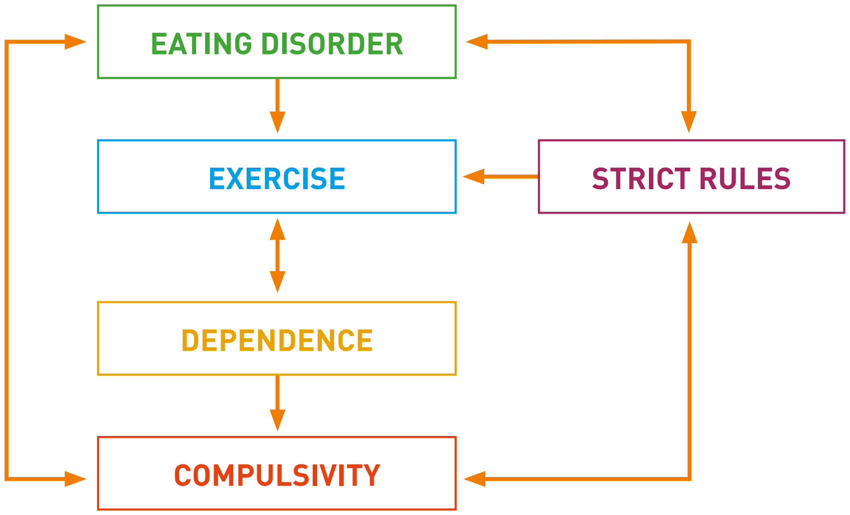

Having an understanding of the maintenance formulation for compulsive exercise will support you in noticing whether your exercise behaviour is healthy or not.

- Is your exercise motivated by weight and shape concerns?
- Is your desire to exercise driven by the positive emotions experienced after it, or to avoid negative emotions?
- Do you have beliefs that make you fearful of stopping exercise?
- Do you follow strict, inflexible rules?

Managing Exercise Urges

Monitoring records bring your attention to your exercise urge and reminds you that you have a choice about engaging in the behaviour or not. They also allow you to take time to challenge your thoughts around the urge to exercise.

Urge surfing is a technique that involves using distraction to resist an urge. If you can resist an urge, you will find that it will become less strong over time, without doing anything other than wait.

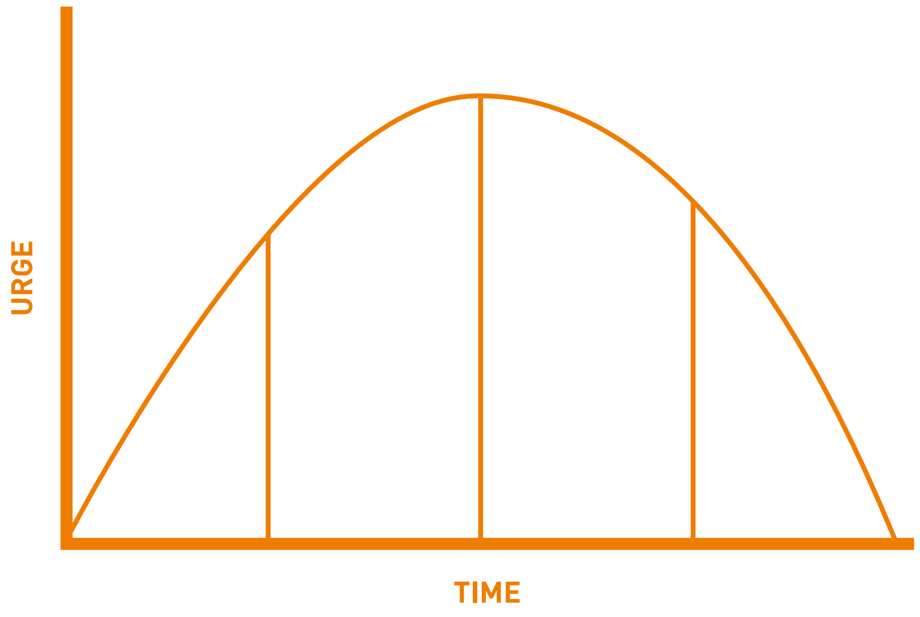

Distraction techniques need to be:

Active you need to be actively engaged in the activity.

Enjoyable it shouldn't feel like a chore.

Realistic it needs to be something that you are able to do. It may be useful to think of different techniques for different situations.

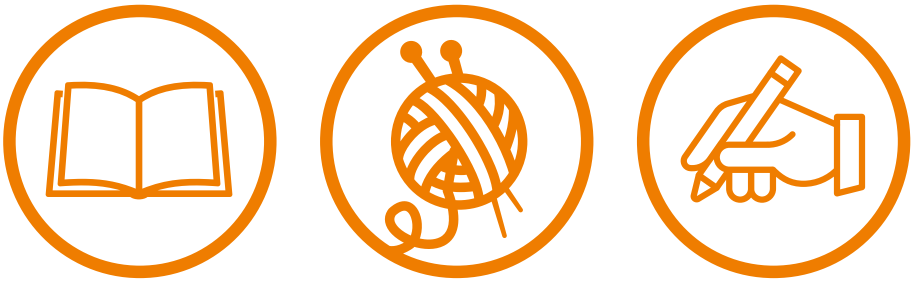

Planning and personalisation is important for distraction!

(resources need to be accessible at the right time)

Session Seven: Review and Reflection

Exercise Maintenance Formulation

Each session of NEAT has focussed on a different section of the maintenance formulation for exercise. This formulation has helped us to break down the different factors that keep exercise going.

Creating your own formulation

Fill in the missing gaps and arrows in the formulation diagram below. In the bubble next to each heading in the formulation, reflect on how this is relevant to you. For example, this could be identifying some of your own strict rules or beliefs that we have thought about in NEAT.

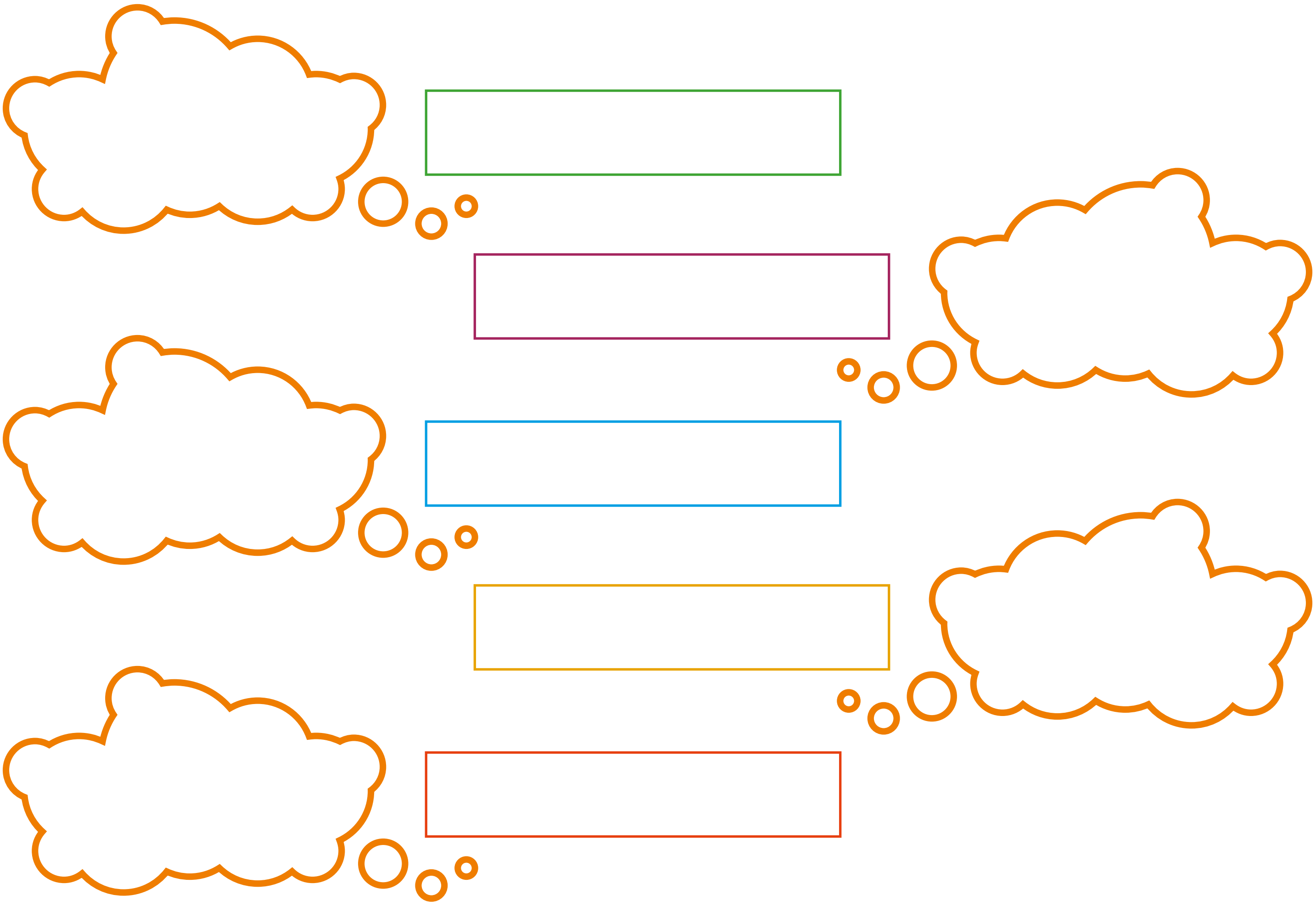

My Exercise Profile

This activity is designed to help you to explore whether there have been any changes in your exercise since you started this group. The questions will help you identify whether your reasons for exercise or the functions exercise serves for you have changed.

Instructions: Read each question carefully and answer it based on the past 2 weeks as honestly as you can. Once you have answered the questions, follow the scoring instructions to calculate your mean (average) score for the scale before progressing to the next one.

Scale 1

If you are feeling low or depressed before you exercise, do you feel any happier (i.e., less depressed or low) after you exercise?	No, never<br>**0**	Yes, a bit happier<br>**1**	Yes, much, much happier<br>**2**
If you are feeling stressed or tense before you exercise, do you feel any less stressed or tense (i.e. more relaxed) after you exercise?	No, never<br>**0**	Yes, a bit less stressed, more relaxed<br>**1**	Yes, totally de-stressed, very relaxed<br>**2**
If you are feeling anxious before you exercise, do you feel any less anxious after you exercise?	No, never<br>**0**	Yes, a bit less anxious<br>**1**	Yes, totally anxiety free<br>**2**
If you have a lot on your mind before you exercise, do you find that the exercise helps you forget your worries/concerns?	No, never<br>**0**	Yes, a little<br>**1**	Yes, totally<br>**2**
Generally, how much of an effect would you say that exercise has on improving your mood (i.e. the way you feel)?	No, effect at all<br>**0**	A reasonable effect<br>**1**	A very large effect<br>**2**

Scoring: Add up (total) each column and add together, then divide the total by 5

**Column Totals:**			
**Grand Total (column totals added together):**			
**Grand Total divided by 5 (Mean Score):**		Round to one decimal place	

Scale 2

If you are unable to exercise, does it make you feel anxious because you cannot exercise?	No, never<br>**0**	Yes, a bit anxious<br>**1**	Yes, very anxious<br>**2**
If you are unable to exercise, do you ever feel frustratedand/or angry because you cannot exercise?	No, never<br>**0**	Sometimes/ occasionally<br>**1**	Usually or always<br>**2**
If you are unable to exercise, does it make you feel upset, low, or depressed because you cannot exercise?	No, never<br>**0**	Yes, a little bit<br>**1**	Yes, very much so<br>**2**
If you are unable to exercise, do you ever feel agitated and/or irritable because you cannot exercise?	No, never<br>**0**	Yes, a little<br>**1**	Usually or always<br>**2**
Do you feel like you need to exercise in order to avoid feeling any or all of the above (i.e., anxious, frustrated, upset, or irritable)?	No, never<br>**0**	A reasonable effect<br>**1**	Yes, very much so<br>**2**

Scoring: Add up (total) each column and add together, then divide the total by 5

**Column Totals:**			
**Grand Total (column totals added together):**			
**Grand Total divided by 5 (Mean Score):**		Round to one decimal place	

Scale 3

Do you try and follow definite rules regarding your exercise for example, a specific number of exercises, or a specific order in which the exercises must be completed?	No, not at all<br>**0**	Sometimes/ occasionally<br>**1**	Usually or always<br>**2**
Do you follow a set routine for your exercise sessions, such as always walk/run the same route, do the same exercises in the same order, spend the same amount of time and so on?	No, never<br>**0**	Sometimes/ occasionally<br>**1**	Usually or always<br>**2**
Would you describe your weekly pattern of exercise as repetitive?	No, not at all<br>**0**	Yes, a little<br>**1**	Yes, very<br>**2**
Do you set yourself exercise goals or targets that you feel you must reach?	No, not at all<br>**0**	Sometimes/ occasionally<br>**1**	Usually or always<br>**2**
Does it upset, annoy or irritate you when your exercise routine is interrupted or you are unable to follow a rule or meet your goals?	No, not at all<br>**0**	Yes, a little<br>**1**	Yes, very<br>**2**

Scoring: Add up (total) each column and add together, then divide the total by 5

**Column Totals:**			
**Grand Total (column totals added together):**			
**Grand Total divided by 5 (Mean Score):**		Round to one decimal place	

Scale 4

If you are unable to exercise, or you miss an exercise session, do you ever feel guilty?	No, never<br>**0**	Yes, a little guilty<br>**1**	Yes, very guilty<br>**2**
Does it worry you if you are unable to exercise, or when you miss an exercise session?	No, never<br>**0**	Yes, a little bit<br>**1**	Yes, a lot<br>**2**
If you miss an exercise session, do you try and make up for it next time you exercise (e.g. by doing more, or putting more effort in)?	No, never<br>**0**	Sometimes/ occasionally<br>**1**	Usually or always<br>**2**
Do you ever feel like you've let yourself down if you miss an exercise session?	No, never<br>**0**	Sometimes/ occasionally<br>**1**	Usually or always<br>**2**
Do you ever make yourself exercise even when you are tired, or do not feel like doing it?	No, never<br>**0**	Sometimes/ occasionally<br>**1**	Usually or always<br>**2**
If you are injured or ill, do you still continue to exercise?	No, never<br>**0**	Sometimes/ occasionally<br>**1**	Usually or always<br>**2**
Does your exercise ever interfere with your social life, work, or your study commitments?	No, never<br>**0**	Sometimes/ occasionally<br>**1**	Usually or always<br>**2**

Scoring: Add up (total) each column and add together, then divide the total by 7

**Column Totals:**			
**Grand Total (column totals added together):**			
**Grand Total divided by 7 (Mean Score):**		Round to one decimal place	

Scale 5

If you are unable to exercise, do you ever worry that you will gain weight, or get fat?	No, never<br>**0**	Sometimes/ occasionally<br>**1**	Usually or always<br>**2**
If you feel that you have eaten too much, do you ever do more exercise to burn it off/make up for it?	No, never<br>**0**	Sometimes/ occasionally<br>**1**	Usually or always<br>**2**
Do you exercise primarily to lose, rather than maintain your weight?	No, not at all<br>**0**	A little<br>**1**	Yes, absolutely<br>**2**
Do you exercise primarily to improve, rather than maintain your appearance and/or shape?	No, not at all<br>**0**	A little<br>**1**	Yes, absolutely<br>**2**

Scoring: Add up (total) each column and add together, then divide the total by 4

**Column Totals:**			
**Grand Total (column totals added together):**			
**Grand Total divided by 4 (Mean Score):**		Round to one decimal place	

Scale 6

	No, not at all	A little	Yes, absolutely
Do you continue to exercise despite finding it a chore?	**0**	**1**	**2**
	Not true at all	A little true	Totally true
How true of you is it to say, "I do not really enjoy exercising"?	**0**	**1**	**2**
	No, not at all	A little	Yes, absolutely
Do you exercise primarily because you feel you have to, or need to, rather than because you enjoy it and want to?	**0**	**1**	**2**

Scoring: Add up (total) each column and add together, then divide the total by 3

**Column Totals:**			
**Grand Total (column totals added together):**			
**Grand Total divided by 3 (Mean Score):**		Round to one decimal place	

My Exercise Profile Graph

Using the mean score that you have calculated for each of the scales, draw a line on the graph and shade the area below the line. For example, if your mean score for scale 1 was 1.6, draw a line at 1.6 in column 1 of the table, and shade the area below the line. Do this for each of the scales.

**2**						
1.9						
1.8						
1.7						
1.6						
1.5						
1.4						
1.3						
1.2						
1.1						
**1**						
0.9						
0.8						
0.7						
0.6						
0.5						
0.4						
0.3						
0.2						
0.1						
**0**						
	Mood Improvement: Positive Reinforcement	Withdrawal Symptoms: Negative Reinforcement (Mood Avoidance)	Behavioural/Exercise Rigidity	Compulsive Exercise	Weight and Shape Exercise	Lack of Exercise Enjoyment
	**Scale 1**	**Scale 2**	**Scale 3**	**Scale 4**	**Scale 5**	**Scale 6**

Reflections of my Exercise Profile

What changes can I see in my exercise profile?

Are there any things that I can continue to work on when looking at my profile?

Why might I still be struggling with these things?

Summary of NEAT

Session 1 Introduction to NEAT

- The maintenance formulation of compulsive exercise.
- The different definitions and the importance of including incidental exercise and physical activity when we think of exercise.

Session 2 Eating Disorder and Exercise

- The different reasons why people exercise.
- The link between eating disorders and exercise and initiating and maintaining factors.
- Looked at activity anorexia and the biological and evolutionary theory to explain the urge of being active at a low weight.

Session 3 Dependence

- Explored exercise dependence and managing mood states.
- Discussed exercising to feel positive emotions or get rid of feeling negative emotions.
- Similarities of exercise being addictive and the withdrawal symptoms that arise, which further increases dependency.

Session 4 Compulsivity

- Beliefs and assumptions which lead to and maintain compulsive exercise.
- Often the compulsivity is driven by fears of what might happen if exercise stopped.
- Challenged these assumptions and whether there is evidence behind them or not.

Session 5 Strict Rules:

- Relationship between eating disorder, behavioural rigidity, and compulsive exercise.
- Perfectionism and black and white thinking are present in eating disorders, therefore there's no deviation from the strict rules.
- Deviating from strict rules also leads to negative emotions which further maintains exercise.
- The differences between healthy and unhealthy rules.

Session 6 Healthy Exercise and Urge Management

- The differences between unhealthy and healthy exercise and ways to identify this.
- Ways of managing exercise urges.

NEAT Exercise Monitoring Record

Use your monitoring record each time you have an exercise urge to record the thoughts and feelings that you experience. Use the final column to report whether you managed to resist the urge and how you managed to do this.

Date and Time	Place / Situation	Desire to exercise 0-100%	Thoughts	Feelings	Outcome How did you manage to resist the urge?	
DD.MM.YY am/pm	Bedroom getting changed for bed	95%	My weight is going to go up after that meal<br>I'm alone, no one will know	Anxious<br>Sad	I didn't<br>Reading<br>Watched TV<br>Listened to music	Other...<br>Made sure I was around people
					I didn't<br>Reading<br>Watched TV<br>Listened to music	Other...
					I didn't<br>Reading<br>Watched TV<br>Listened to music	Other...
					I didn't<br>Reading<br>Watched TV<br>Listened to music	Other...
					I didn't<br>Reading<br>Watched TV<br>Listened to music	Other...
					I didn't<br>Reading<br>Watched TV<br>Listened to music	Other...

NEAT Activity Questionnaire

Date:..

What was todays activity?

....

Would you consider this to be a low or a high intensity activity?

....

Do you think that today's session has been a satisfactory amount of activity for your week?

....

Did you experience any urges to do more than recommended in the session? (please give details)

....

....

On a scale or 1 – 10 how would you have rated your mood at the beginning of the session? (1 = low / anxious, 10 = happy / relaxed)

1 2 3 4 5 6 7 8 9 10

On a scale or 1 – 10 how would you rate your mood at the end of the session? (1 = low / anxious, 10 = happy / relaxed)

1 2 3 4 5 6 7 8 9 10

Any other reflections on your thoughts and feelings in today's session?

....

....

Activity Anorexia Quiz

1. What must you do to enable your Leptin levels to return to normal?

- [ ] Maintain your weight
- [ ] Eat before exercise
- [ ] Return to a 'normal' bodyweight

2. How does your body initially respond to a fall in bodyweight?

- [ ] Decreases energy levels
- [ ] Increases energy levels
- [ ] Maintain energy levels

3. What happened to the exercising rats that had their diet restricted?

- [ ] They exercised last
- [ ] They were the healthiest rats
- [ ] They ran themselves to death

4. Why does the body increase energy levels in response to a fall in bodyweight?

- [ ] It is more effective
- [ ] It is an evolutionary survival mechanism
- [ ] The thinner you are, the fitter you are

5. What effect does the increase in energy levels due to low bodyweight have on the body?

- [ ] It places considerable stress on the body
- [ ] It has no effect
- [ ] It mildly stresses the body

6. What is the best way to manage the increase in energy levels?

- [ ] Exercise until tired
- [ ] Keep yourself busy
- [ ] Restrict activity and normalise bodyweight

SCORE: ....out of six

Exercise Profile

Using the mean score that you have calculated for each of the scales, draw a line on the graph and shade the area below the line. For example, if your mean score for scale 1 was 1.6, draw a line at 1.6 in column 1 of the table, and shade the area below the line. Do this for each of the scales.

**2**						
1.9						
1.8						
1.7						
1.6						
1.5						
1.4						
1.3						
1.2						
1.1						
**1**						
0.9						
0.8						
0.7						
0.6						
0.5						
0.4						
0.3						
0.2						
0.1						
**0**						
	Mood Improvement: Positive Reinforcement	Withdrawal Symptoms: Negative Reinforcement (Mood Avoidance)	Behavioural/Exercise Rigidity	Compulsive Exercise	Weight and Shape Exercise	Lack of Exercise Enjoyment
	**Scale 1**	**Scale 2**	**Scale 3**	**Scale 4**	**Scale 5**	**Scale 6**

Sincere thanks to all the staff and patients for all their hard work and dedication during the development of this programme.

The young people treated at Newbridge have played an integral role evaluating the programme and through this, it has been shaped and refined. Like all our interventions, the commitment and dedication of Newbridge staff and the involvement of young people have been essential to its development, impact and success.
